# Supplementary material for: FOCS: a novel method for analyzing enhancer and gene activity patterns infers an extensive enhancer–promoter map
Source: Genome Biol. 2018 May 1;19:56. doi: 10.1186/s13059-018-1432-2 (PMC5930446; doi:10.1186/s13059-018-1432-2)
Supplement: Supplementary file 1 — Figures S1–S14, Tables S1–S3, and Supplemental Methods. (PDF 3127 kb) [file 13059_2018_1432_MOESM1_ESM.pdf]

This PDF includes:

- Supplementary Figures 1-14
- Supplementary Tables 1-3
- Supplemental Methods

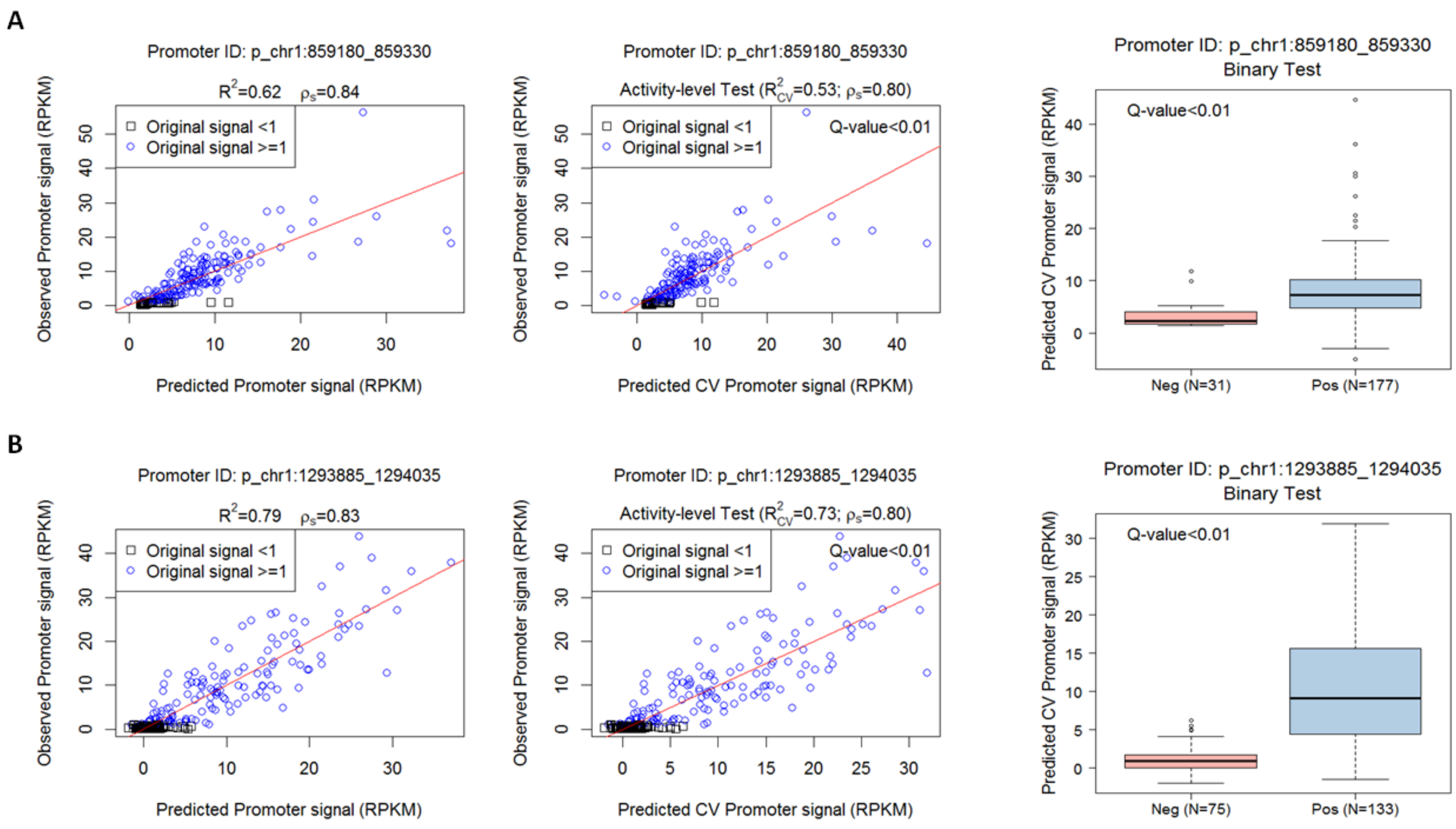
A
Promoter ID: p_chr1:859180_859330
R2=0.62 ρs=0.84
Original signal <1
Original signal >=1
Observed Promoter signal (RPKM)
Predicted Promoter signal (RPKM)
Activity-level Test (R2CV=0.53; ρs=0.80)
Q-value<0.01
Predicted CV Promoter signal (RPKM)
Binary Test
Neg (N=31)
Pos (N=177)
B
Promoter ID: p_chr1:1293885_1294035
R2=0.79 ρs=0.83
Activity-level Test (R2CV=0.73; ρs=0.80)
Neg (N=75)
Pos (N=133)

Fig. S1

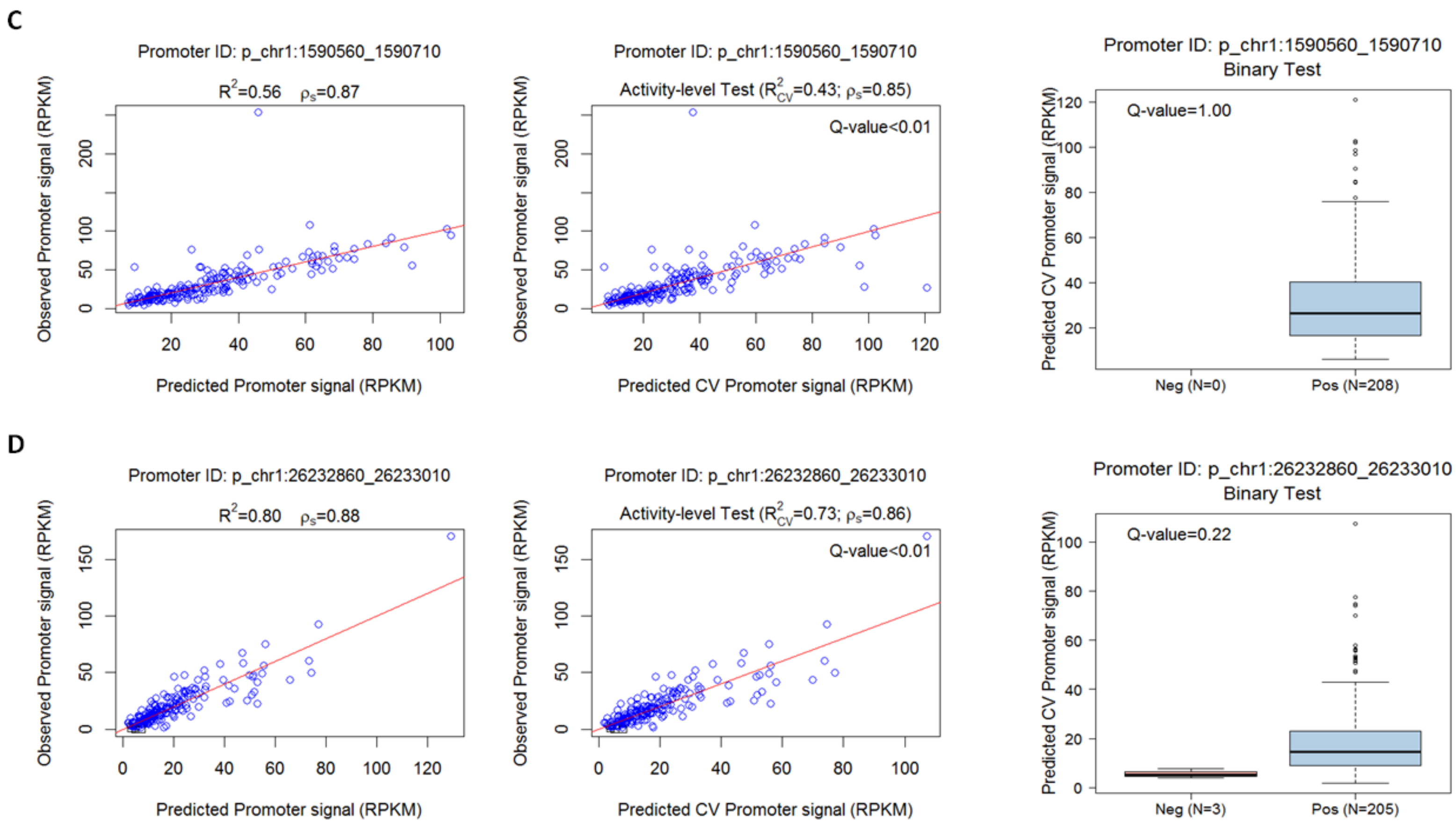
C
Promoter ID: p_chr1:1590560_1590710
R2=0.56 ρs=0.87
Observed Promoter signal (RPKM)
Predicted Promoter signal (RPKM)
Promoter ID: p_chr1:1590560_1590710
Activity-level Test (R2CV=0.43; ρs=0.85)
Q-value<0.01
Observed Promoter signal (RPKM)
Predicted CV Promoter signal (RPKM)
Promoter ID: p_chr1:1590560_1590710
Binary Test
Q-value=1.00
Predicted CV Promoter signal (RPKM)
Neg (N=0)
Pos (N=208)
D
Promoter ID: p_chr1:26232860_26233010
R2=0.80 ρs=0.88
Observed Promoter signal (RPKM)
Predicted Promoter signal (RPKM)
Promoter ID: p_chr1:26232860_26233010
Activity-level Test (R2CV=0.73; ρs=0.86)
Q-value<0.01
Observed Promoter signal (RPKM)
Predicted CV Promoter signal (RPKM)
Promoter ID: p_chr1:26232860_26233010
Binary Test
Q-value=0.22
Predicted CV Promoter signal (RPKM)
Neg (N=3)
Pos (N=205)

Fig. S1

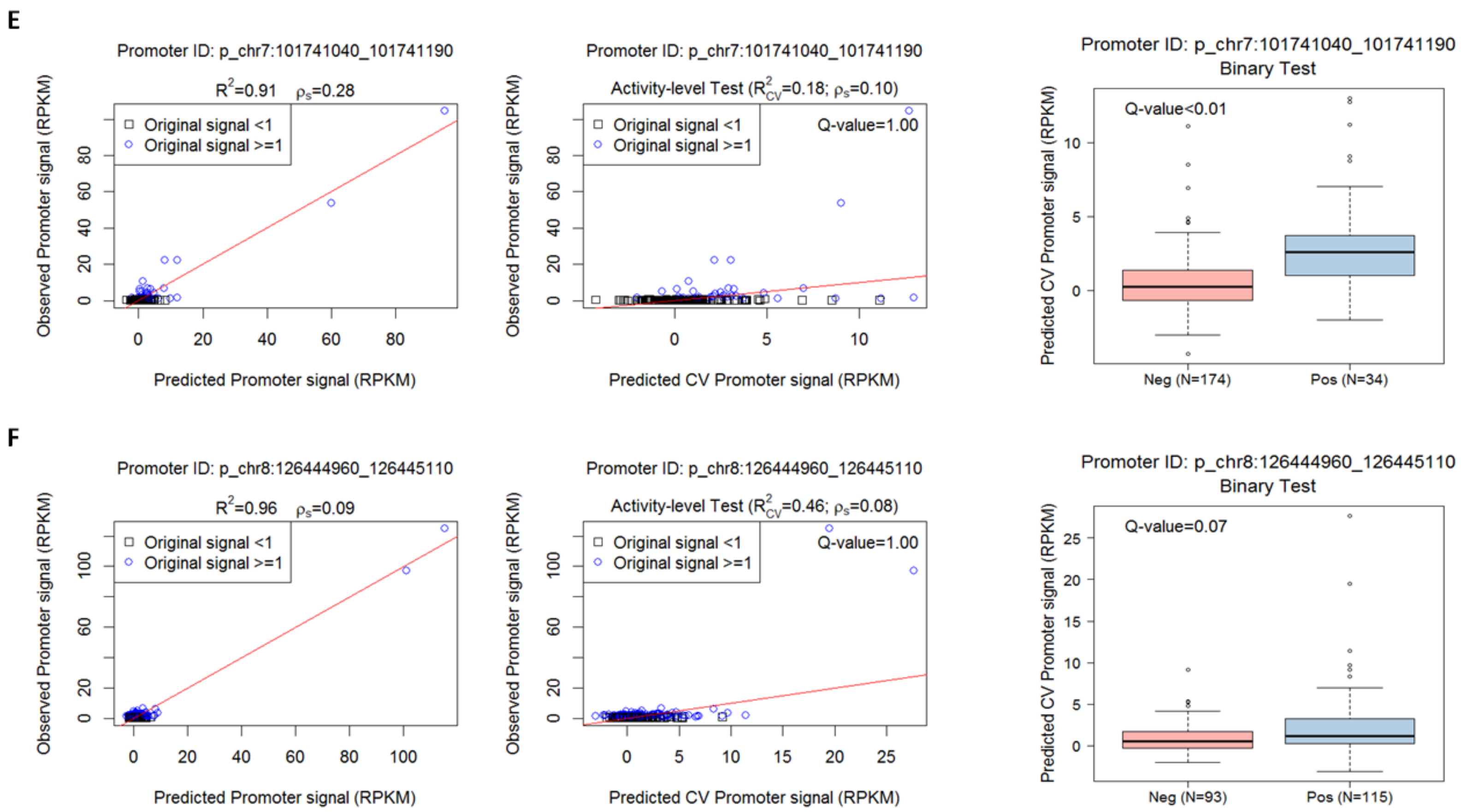
E
Promoter ID: p_chr7:101741040_101741190
R2=0.91 ρs=0.28
Original signal <1
Original signal >=1
Observed Promoter signal (RPKM)
Predicted Promoter signal (RPKM)
Activity-level Test (R2CV=0.18; ρs=0.10)
Q-value=1.00
Predicted CV Promoter signal (RPKM)
Binary Test
Q-value<0.01
Neg (N=174)
Pos (N=34)
F
Promoter ID: p_chr8:126444960_126445110
R2=0.96 ρs=0.09
Activity-level Test (R2CV=0.46; ρs=0.08)
Q-value=1.00
Q-value=0.07
Neg (N=93)
Pos (N=115)

Fig. S1

Supplementary Figure 1. Examples of cross-validated promoter models. Examples of promoter models that passed one or both cross-validation tests: (A-B) passed both binary and level tests (C-D) passed only the activity level test and (E-F) passed only the binary test. For each promoter, the left panel shows the correlation between observed and predicted promoter activities using OLS without cross-validation; the middle panel shows the results of the activity level validation test. Namely, the correlation between observed activities and activities that were predicted on left-out samples (LCTO CV procedure). In this test, correlation is calculated only over positive samples. The right panel shows the results of the binary test. Note in E and F left panel, the sensitivity of R^2 (and, equally, of Pearson correlation) to outliers.

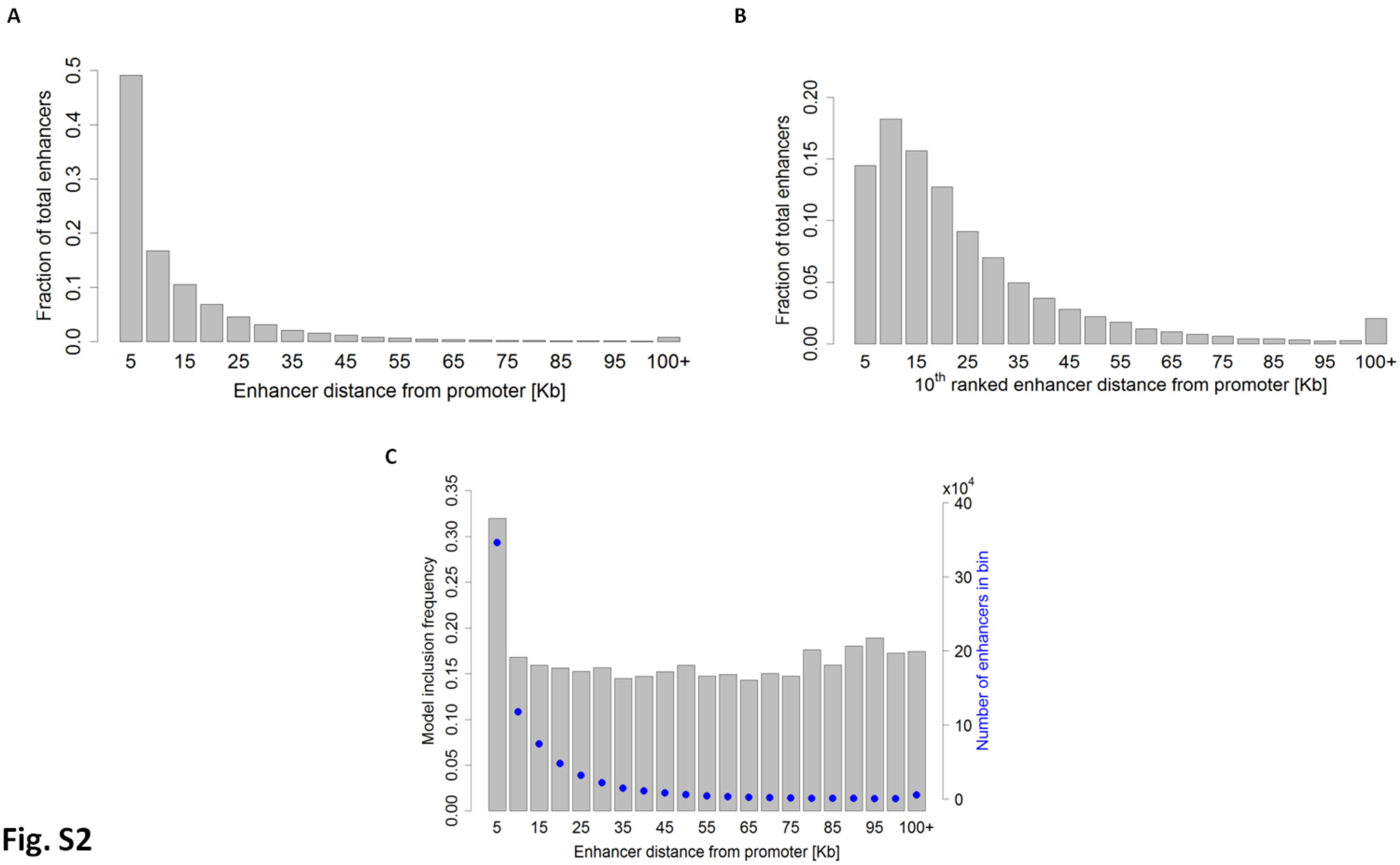
A
Fraction of total enhancers
0.0
0.1
0.2
0.3
0.4
0.5
5
15
25
35
45
55
65
75
85
95
100+
Enhancer distance from promoter [Kb]
B
Fraction of total enhancers
0.00
0.05
0.10
0.15
0.20
10th ranked enhancer distance from promoter [Kb]
C
Model inclusion frequency
0.00
0.05
0.10
0.15
0.20
0.25
0.30
0.35
x10^4
0
10
20
30
40
Number of enhancers in bin
Enhancer distance from promoter [Kb]

Fig. S2

Supplementary Figure 2. E-P distance distribution. E-P distance distribution for: (A). All 10 enhancers in the models that passed cross validation. (B). The 10th enhancer (ranked by distance to promoter) in the models that passed cross validation. (C). Enhancer inclusion frequency in the optimally reduced models. Blue dots denote the total number of enhancers (right y-axis) in each distance bin before the shrinkage step.

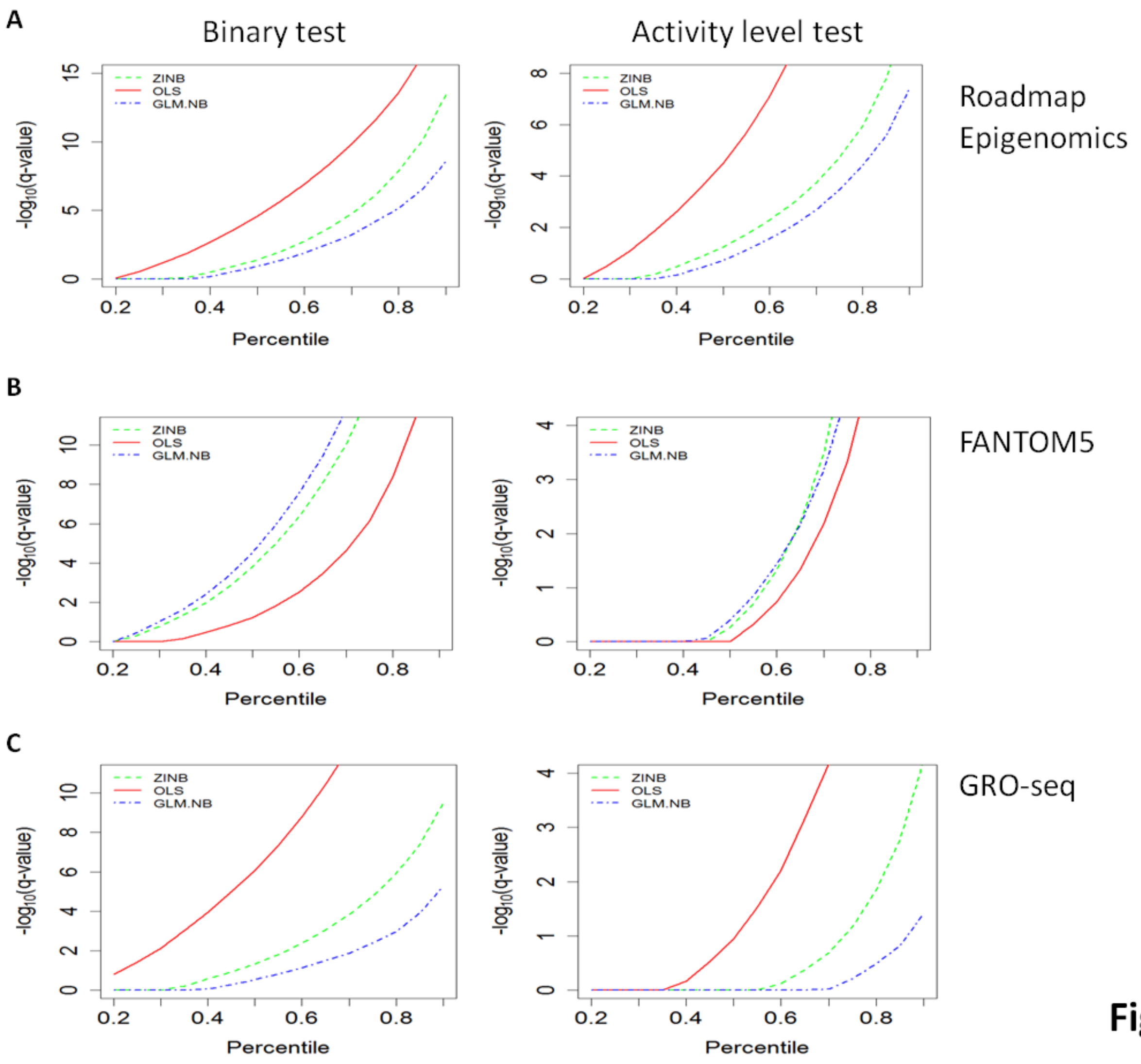
A
Binary test
Activity level test
Roadmap Epigenomics
B
FANTOM5
C
GRO-seq
ZINB
OLS
GLM.NB
$-\log_{10}$(q-value)
Percentile

Fig. S3

Supplementary Figure 3. Performance of three alternative regression methods for inferring E-P models. Same as Figure 2A-B, but here analysis was applied to Roadmap Epigenomics (A), FANTOM5 (B) and the GRO-seq (C) datasets. Results of the binary (left panel) and activity level (right panel) validation tests are shown. OLS performed better on the Roadmap Epigenomics and GRO-seq datasets (in addition to the ENCODE data (Fig. 2A-B)), while GLM.NB and ZINB performed better on the FANTOM5 dataset.

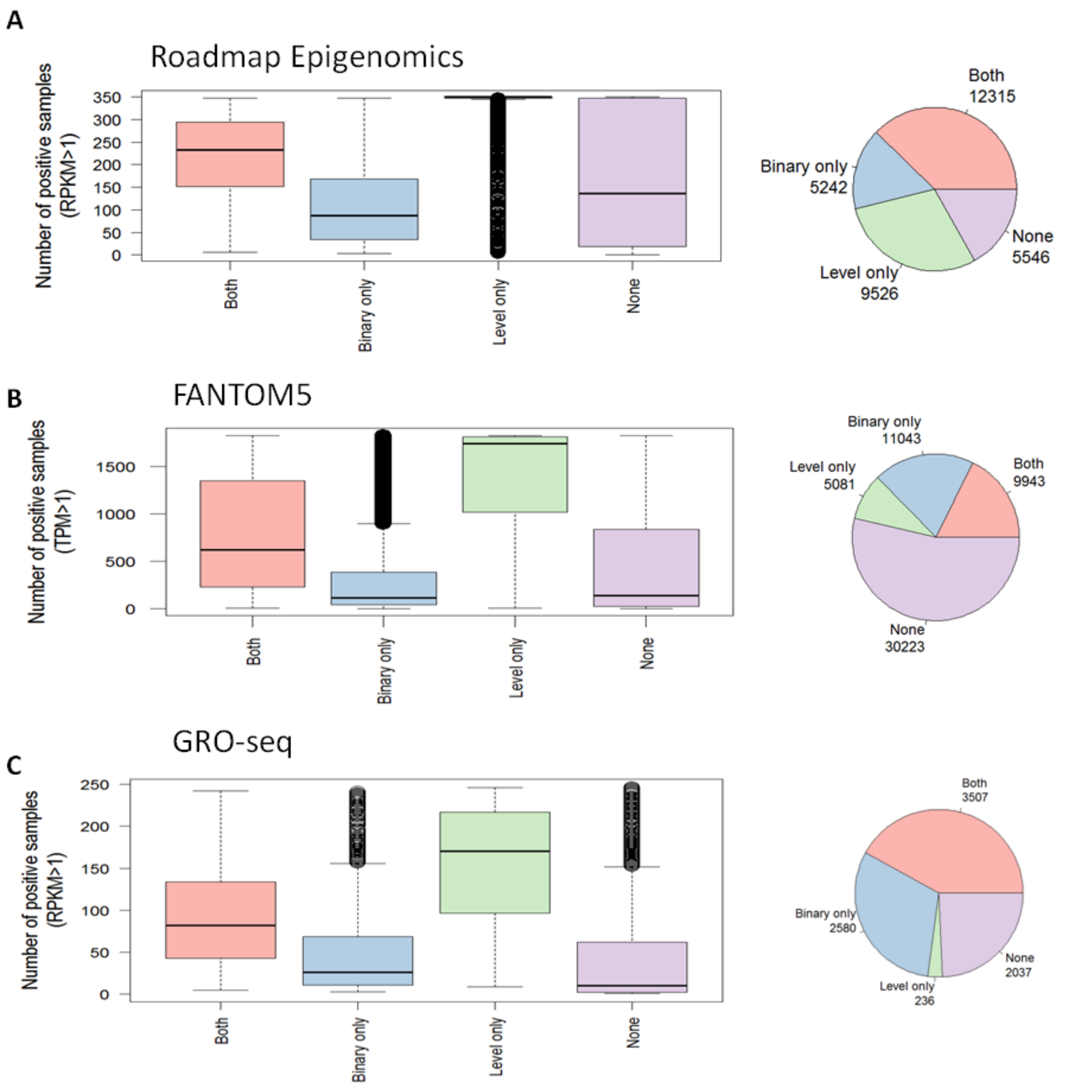
A
Roadmap Epigenomics
Number of positive samples (RPKM>1)
350
300
250
200
150
100
50
0
Both
Binary only
Level only
None
Both 12315
Binary only 5242
None 5546
Level only 9526
B
FANTOM5
Number of positive samples (TPM>1)
1500
1000
500
0
Both
Binary only
Level only
None
Binary only 11043
Level only 5081
Both 9943
None 30223
C
GRO-seq
Number of positive samples (RPKM>1)
250
200
150
100
50
0
Both
Binary only
Level only
None
Both 3507
Binary only 2580
None 2037
Level only 236

Fig. S4

Supplementary Figure 4. Number of validated promoter models. Number of promoters whose OLS models passed (at q-value<0.1) each of the validation tests (right panel) and the distribution of the number of positive samples in each category. (A). Roadmap Epigenomics; (B) FANTOM5 and (C) GRO-seq datasets.

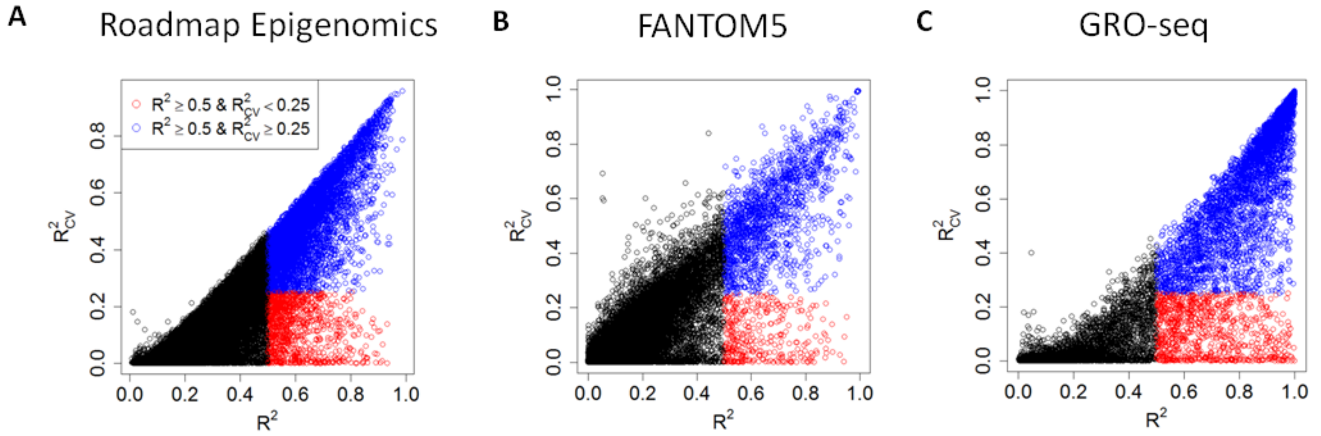
A
Roadmap Epigenomics
B
FANTOM5
C
GRO-seq
$R^2 \geq 0.5$ & $R^2_{CV} < 0.25$
$R^2 \geq 0.5$ & $R^2_{CV} \geq 0.25$
R^2_{CV}
R^2
0.0
0.2
0.4
0.6
0.8
1.0

Fig. S5

Supplementary Figure 5. **Comparison between the R^2 values with and without cross-validation (CV)**. (A). Roadmap Epigenomics; (B) FANTOM5 and (C) GRO-seq datasets. Each dot is a promoter model. Blue dots denote models with $R^2 \geq 0.5$ and $R_{CV}^2 \geq 0.25$. Red dots denote models with and $R^2 > 0.5$ and $R_{CV}^2 < 0.25$. The high rate of red dots (Roadmap (16%), FANTOM5 (20%) and GRO-seq (22%)) indicates that training the models on all samples suffer from overfitting.

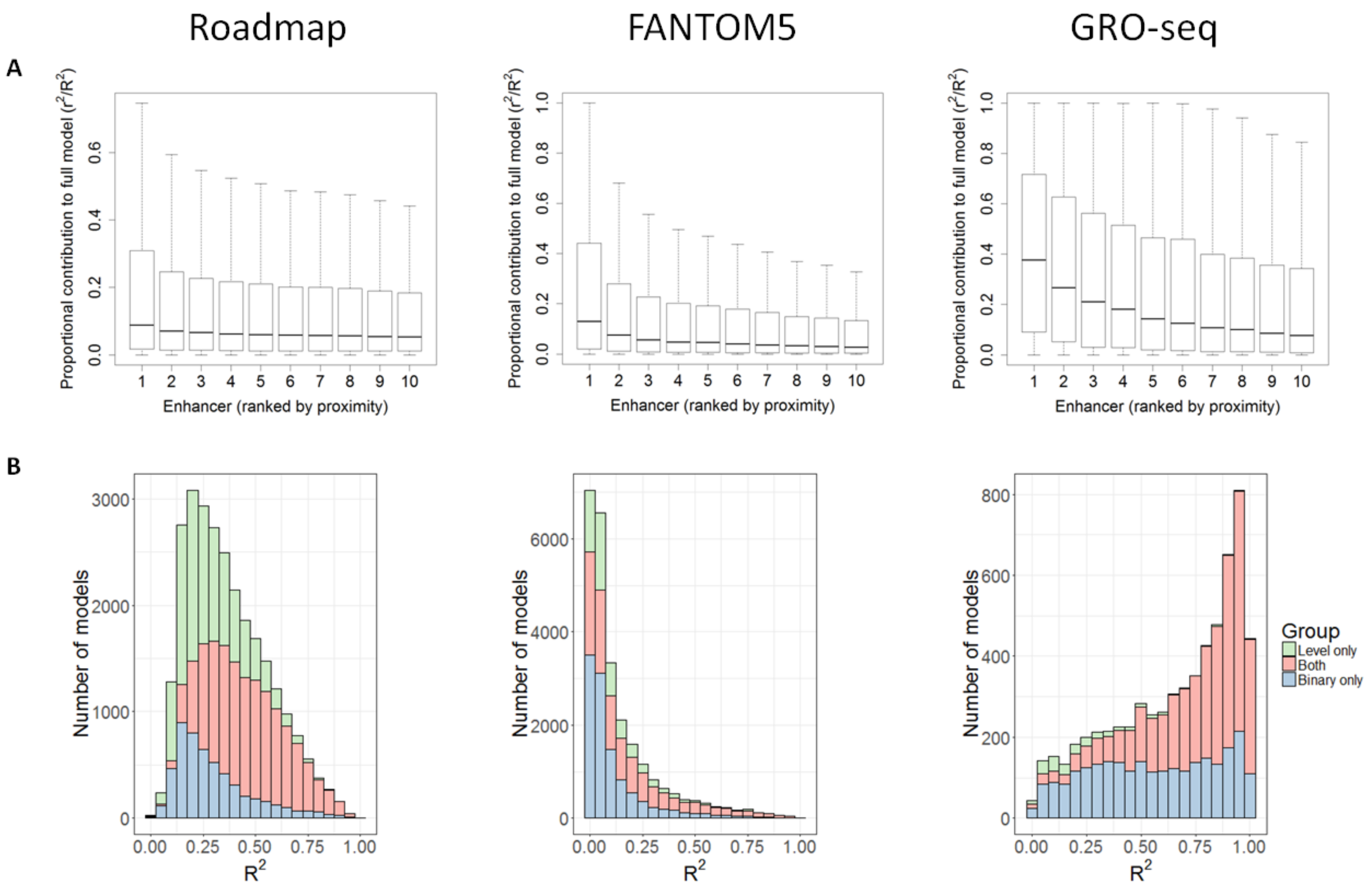
Roadmap
FANTOM5
GRO-seq
A
Proportional contribution to full model (r²/R²)
Enhancer (ranked by proximity)
B
Number of models
R²
Group
Level only
Both
Binary only

Fig. S6

Supplementary Figure 6. Configuration of promoter regulation by enhancers. (A). The proportional contribution of the 10 most proximal enhancers (within a distance of ±500kb from the target promoter; for FANTOM5 the distance was ±250kb from the target promoter) to the regression model, in each dataset (Roadmap Epigenomics, FANTOM5 and GRO-seq). The X axis indicates the order of the enhancers by their relative distance from the promoter, with 1 being the closest. (B) R^2 values of the models that passed one or both CV tests, in each dataset.

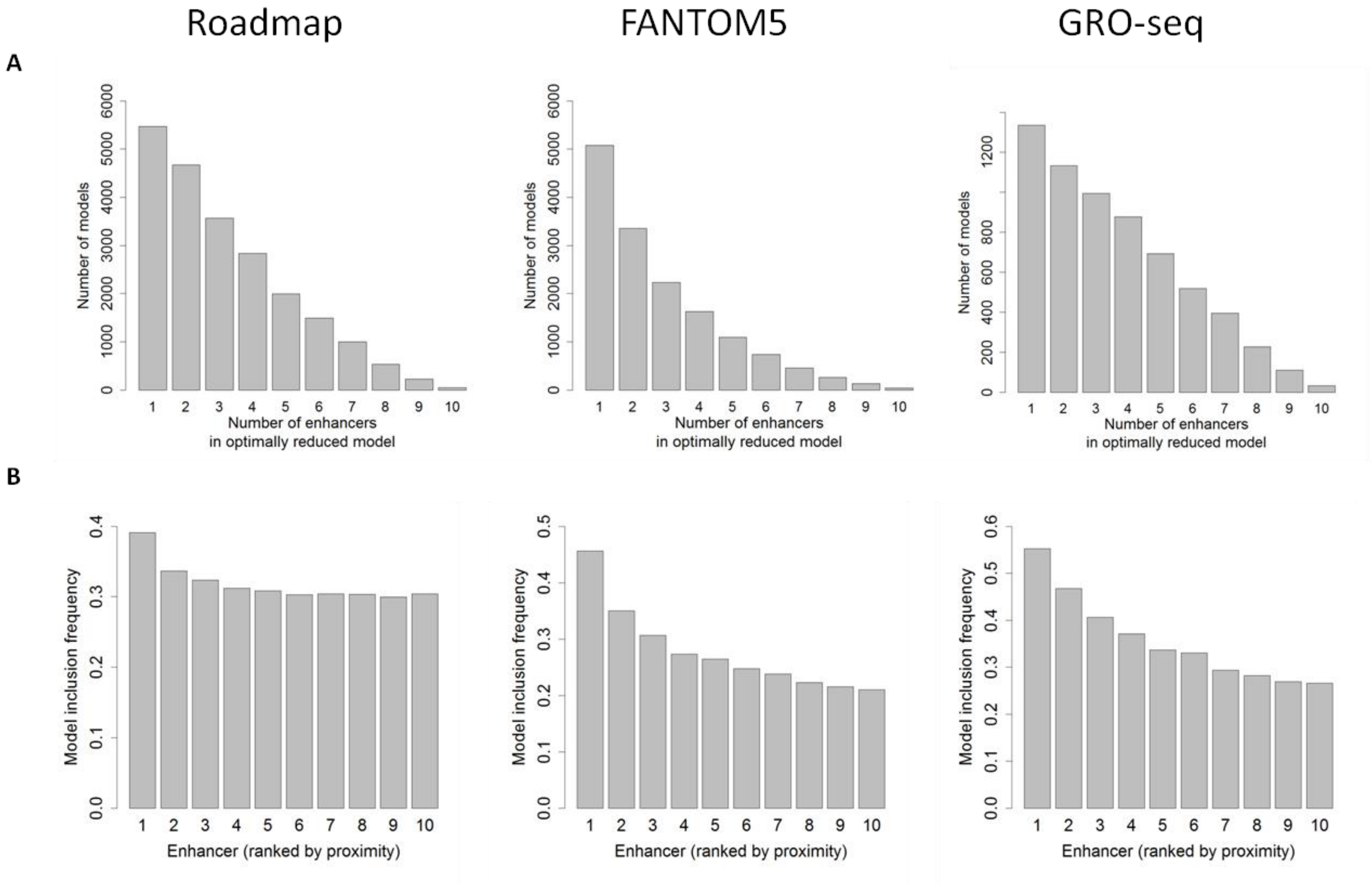

Roadmap
FANTOM5
GRO-seq
A
Number of models
Number of enhancers in optimally reduced model
B
Model inclusion frequency
Enhancer (ranked by proximity)

Fig. S7

Supplementary Figure 7. Configuration of shrunken promoter models. (A) Distribution of the number of enhancers included in the validated, optimally-reduced models (i.e. after elastic net shrinkage). (B) Inclusion frequency of enhancers in the reduced models as a function of their proximity ranking to the target promoter.

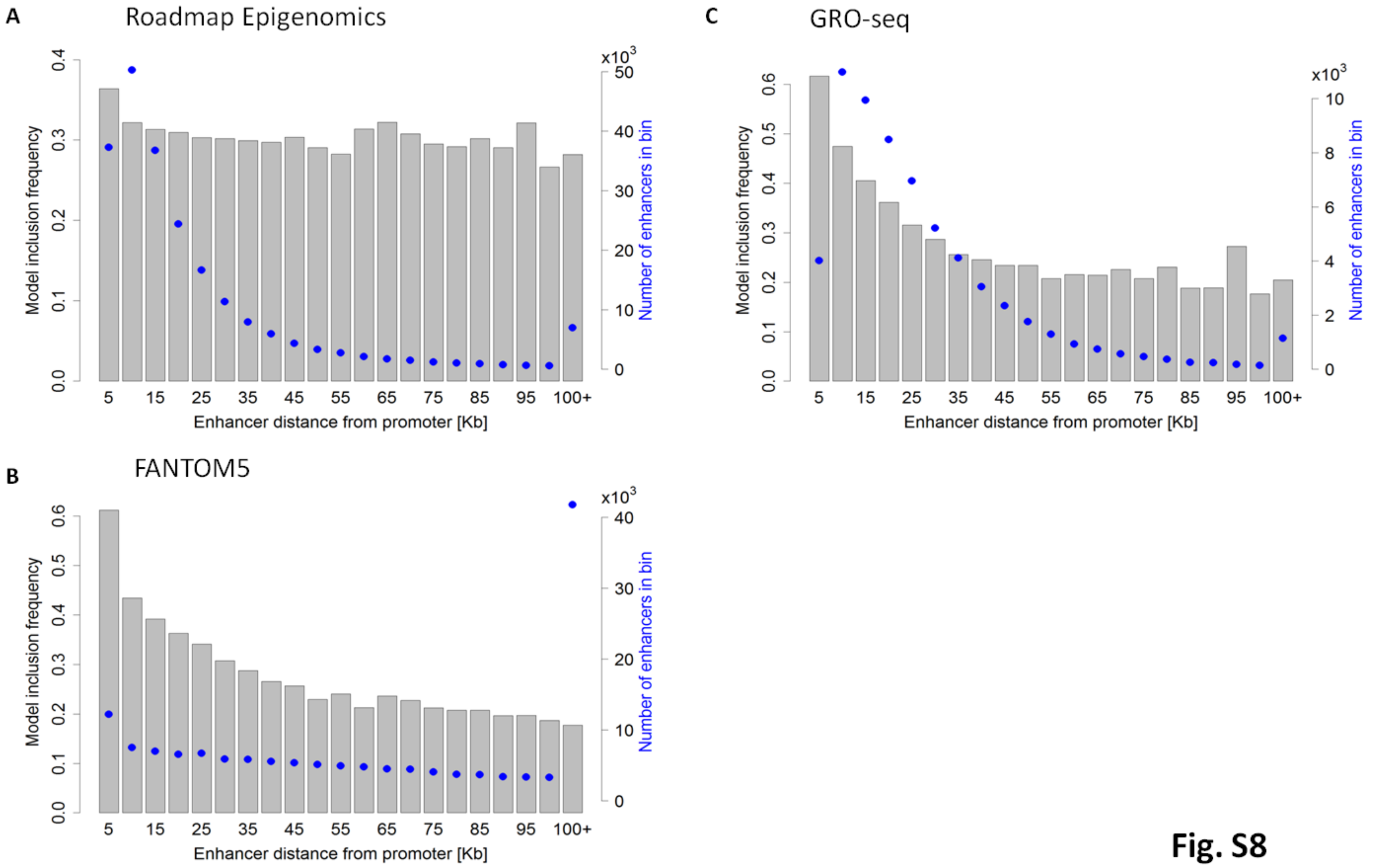
A
Roadmap Epigenomics
Model inclusion frequency
0.0
0.1
0.2
0.3
0.4
x10³
0
10
20
30
40
50
Number of enhancers in bin
5
15
25
35
45
55
65
75
85
95
100+
Enhancer distance from promoter [Kb]
B
FANTOM5
Model inclusion frequency
0.0
0.1
0.2
0.3
0.4
0.5
0.6
x10³
0
10
20
30
40
Number of enhancers in bin
5
15
25
35
45
55
65
75
85
95
100+
Enhancer distance from promoter [Kb]
C
GRO-seq
Model inclusion frequency
0.0
0.1
0.2
0.3
0.4
0.5
0.6
x10³
0
2
4
6
8
10
Number of enhancers in bin
5
15
25
35
45
55
65
75
85
95
100+
Enhancer distance from promoter [Kb]

Fig. S8

Supplementary Figure 8. Inclusion frequency of enhancers as function of E-P distance. Inclusion frequency of enhancers in the reduced models as a function of their distance from the target promoter for (A) Roadmap Epigenomics, (B) FANTOM5 and (C) GRO-seq datasets. Blue dots denote the number of enhancers (right y-axis) in each bin before the shrinkage step.

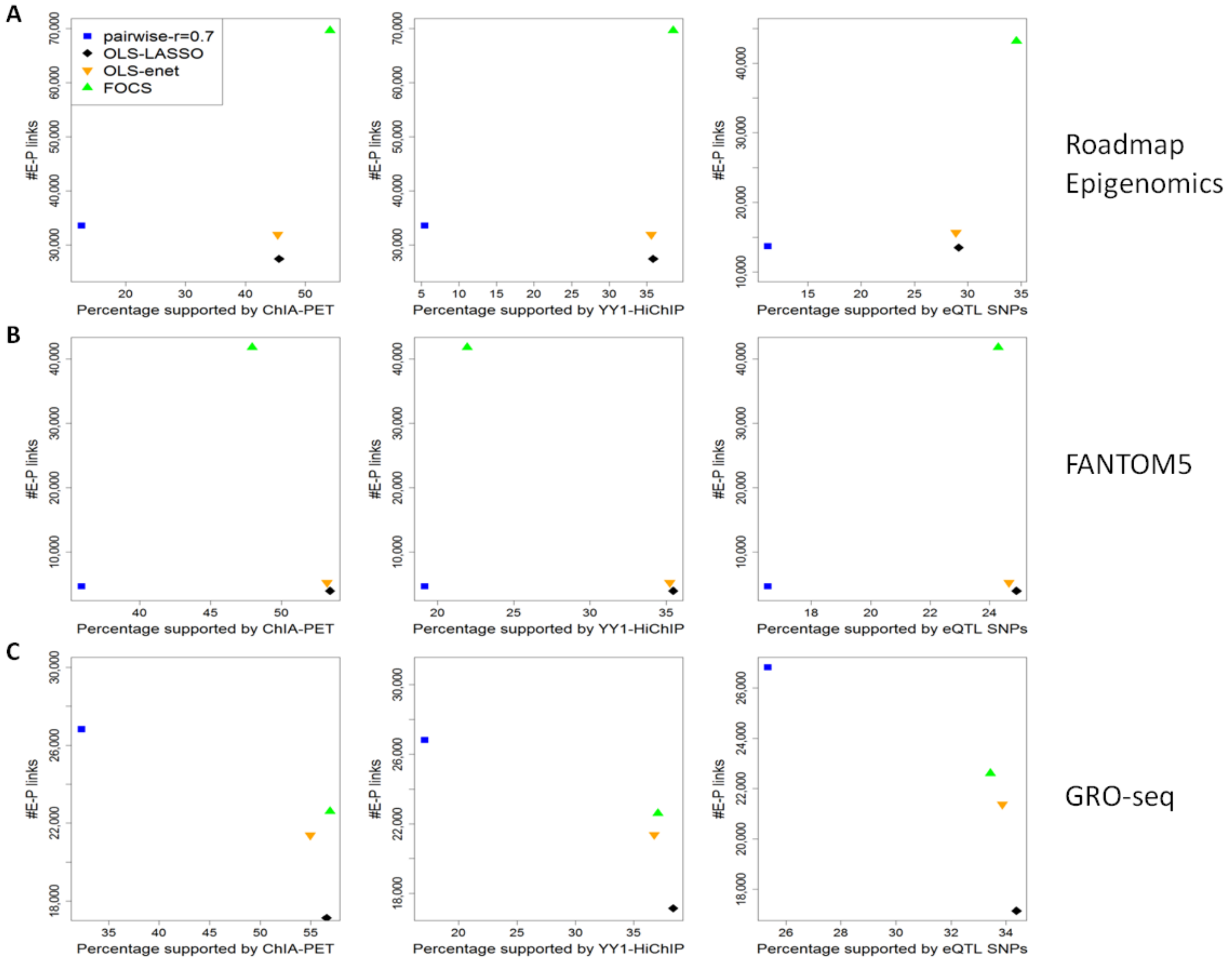
A
B
C
pairwise-r=0.7
OLS-LASSO
OLS-enet
FOCS
#E-P links
Percentage supported by ChIA-PET
Percentage supported by YY1-HiChIP
Percentage supported by eQTL SNPs
Roadmap Epigenomics
FANTOM5
GRO-seq

Fig. S9

Supplementary Figure 9. Comparison of the performance of different methods for predicting E-P links using ChIA-PET, YY1-HiChIP and eQTL data as external validation. As in Fig. 4, but for Roadmap Epigenomics (A), FANTOM5 (B) and GRO-seq (C) datasets.

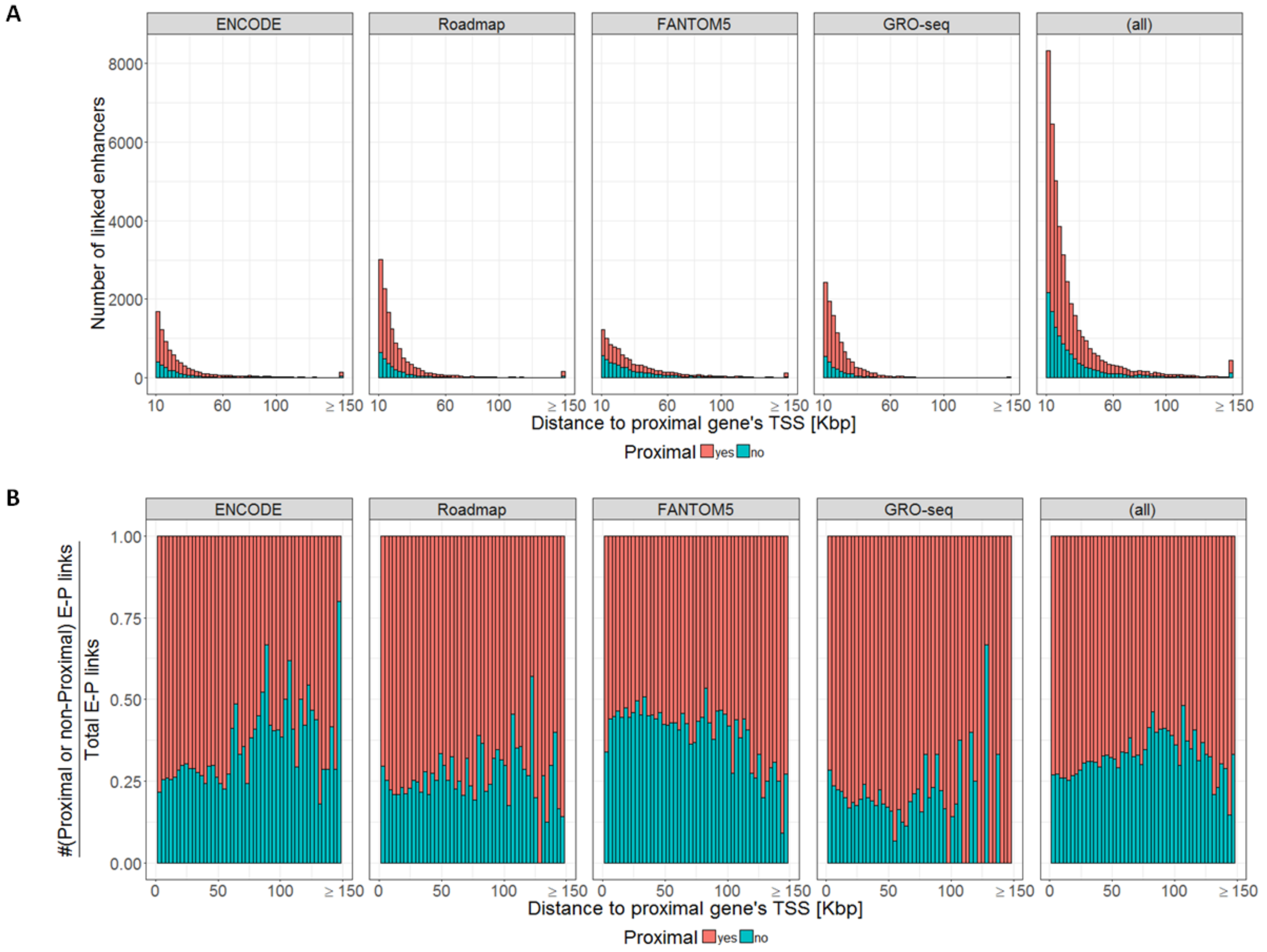
A
ENCODE
Roadmap
FANTOM5
GRO-seq
(all)
Number of linked enhancers
8000
6000
4000
2000
0
10
60
100
≥ 150
Distance to proximal gene's TSS [Kbp]
Proximal yes no
B
#(Proximal or non-Proximal) E-P links
Total E-P links
1.00
0.75
0.50
0.25
0.00
0
50
100
≥ 150
Distance to proximal gene's TSS [Kbp]
Proximal yes no

Fig. S10

Supplementary Figure 10. Enhancers are frequently linked to genes more distal to the nearest one. The number (A) and proportion (B) of enhancers that are linked to nearest/more distal promoter as a function of their distance to the nearest promoter.

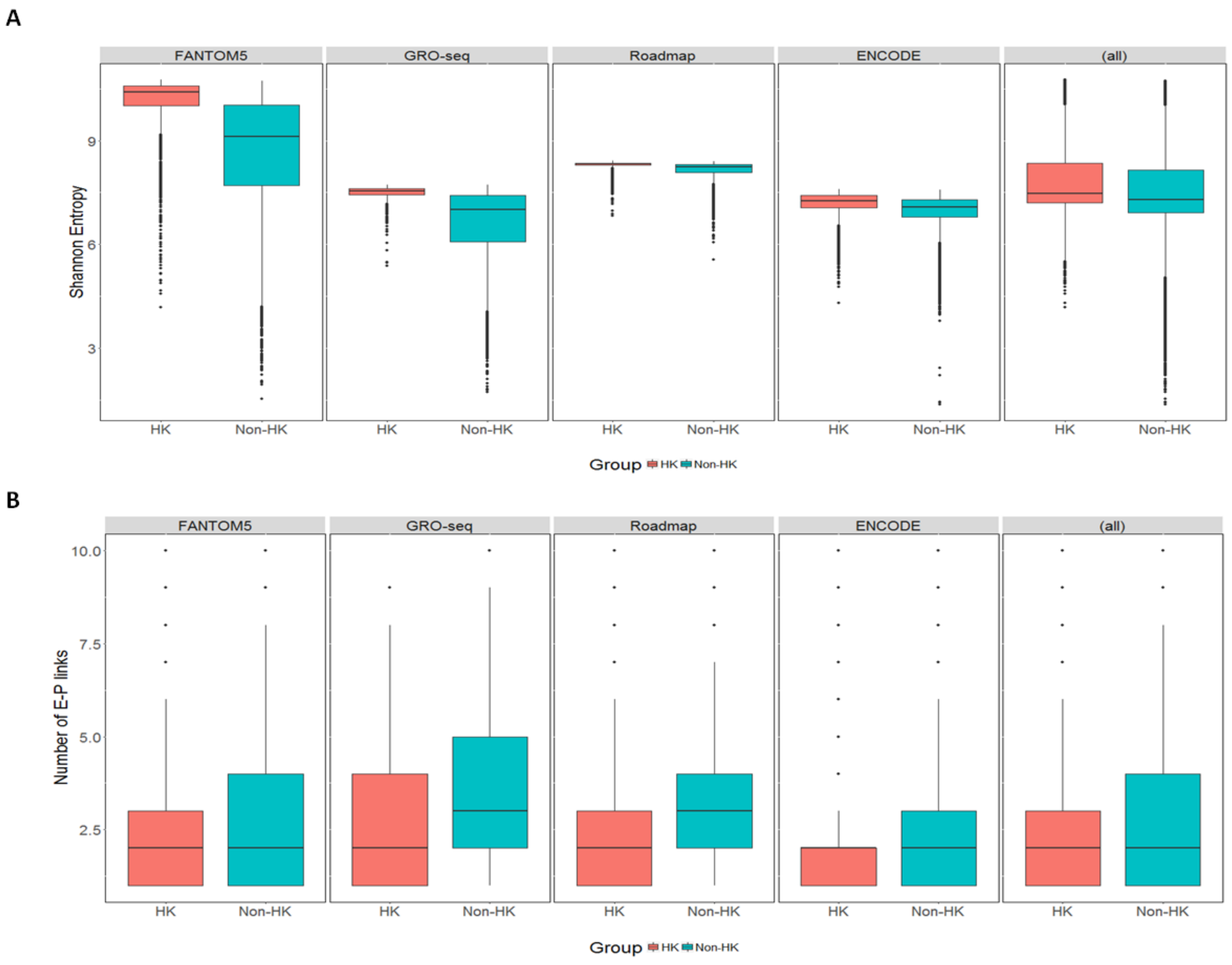
A
FANTOM5
GRO-seq
Roadmap
ENCODE
(all)
Shannon Entropy
9
6
3
HK
Non-HK
Group HK Non-HK
B
FANTOM5
GRO-seq
Roadmap
ENCODE
(all)
Number of E-P links
10.0
7.5
5.0
2.5
HK
Non-HK
Group HK Non-HK

Fig. S11

Supplementary Figure 11. House-keeping genes show simpler pattern of E-P interactions. (A). Ubiquitous vs. cell-type specific expression pattern is quantified by Shannon Entropy. In all datasets, housekeeping (HK) genes show significantly higher Shannon Entropy than the rest of genes, reflecting their more uniform activity pattern over the examined cell panel. (B). Promoters of HK genes are involved in significantly lower number of E-P interactions than other genes (in all cases, p-value << 0.001; calculated by one-sided Wilcoxon rank-sum test).

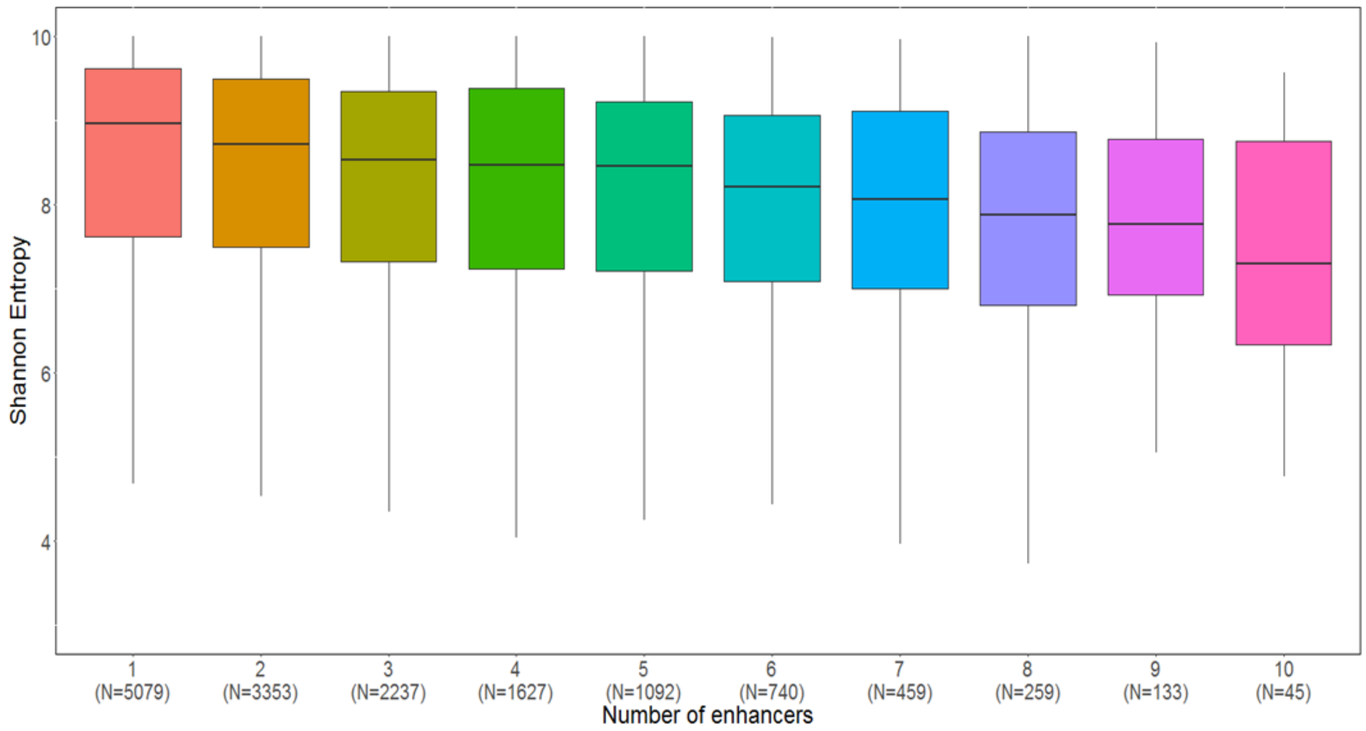

Fig. S12

Supplementary Figure 12. Opposite relationship between breadth of promoter activity over cell types and complexity of transcriptional regulation. Same analysis as shown in Fig. 6, but here applied to FANTOM5 CAGE data.

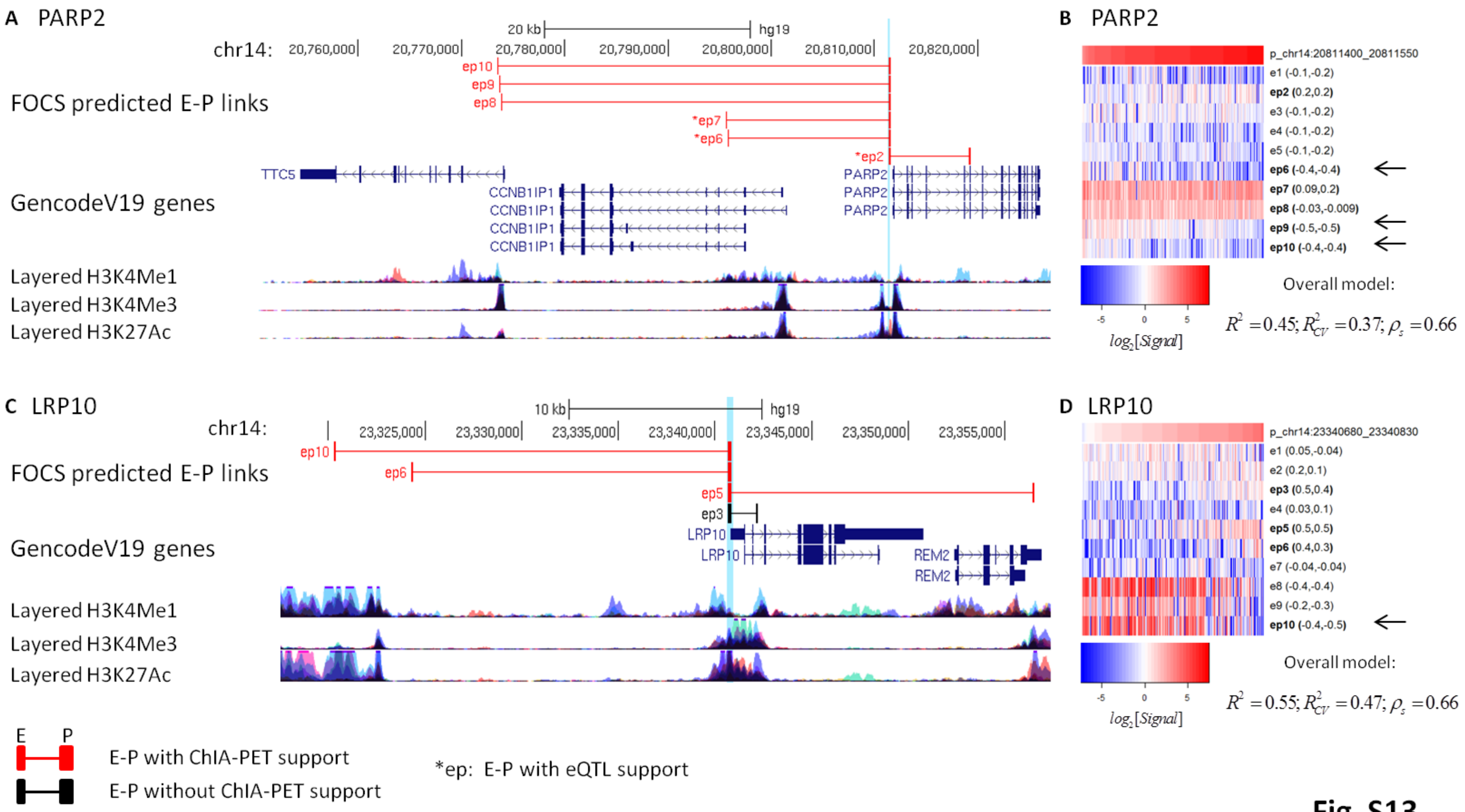

A PARP2
chr14:
20 kb
hg19
20,760,000
20,770,000
20,780,000
20,790,000
20,800,000
20,810,000
20,820,000
FOCS predicted E-P links
ep10
ep9
ep8
*ep7
*ep6
*ep2
GencodeV19 genes
TTC5
CCNB1IP1
PARP2
Layered H3K4Me1
Layered H3K4Me3
Layered H3K27Ac
B PARP2
p_chr14:20811400_20811550
e1 (-0.1,-0.2)
ep2 (0.2,0.2)
e3 (-0.1,-0.2)
e4 (-0.1,-0.2)
e5 (-0.1,-0.2)
ep6 (-0.4,-0.4)
ep7 (0.09,0.2)
ep8 (-0.03,-0.009)
ep9 (-0.5,-0.5)
ep10 (-0.4,-0.4)
-5
0
5
log2[Signal]
Overall model:
$R^2 = 0.45; R^2_{CV} = 0.37; \rho_s = 0.66$
C LRP10
chr14:
10 kb
hg19
23,325,000
23,330,000
23,335,000
23,340,000
23,345,000
23,350,000
23,355,000
FOCS predicted E-P links
ep10
ep6
ep5
ep3
GencodeV19 genes
LRP10
REM2
Layered H3K4Me1
Layered H3K4Me3
Layered H3K27Ac
D LRP10
p_chr14:23340680_23340830
e1 (0.05,-0.04)
e2 (0.2,0.1)
ep3 (0.5,0.4)
e4 (0.03,0.1)
ep5 (0.5,0.5)
ep6 (0.4,0.3)
e7 (-0.04,-0.04)
e8 (-0.4,-0.4)
e9 (-0.2,-0.3)
ep10 (-0.4,-0.5)
-5
0
5
log2[Signal]
Overall model:
$R^2 = 0.55; R^2_{CV} = 0.47; \rho_s = 0.66$
E
P
E-P with ChIA-PET support
E-P without ChIA-PET support
*ep: E-P with eQTL support

Fig. S13

Supplementary Figure 13. Examples for promoter models that include negatively correlated enhancers. (see legend of Fig. 5). In the heatmap, negatively correlated enhancers (indication of a repressor function) are indicated by an arrow.

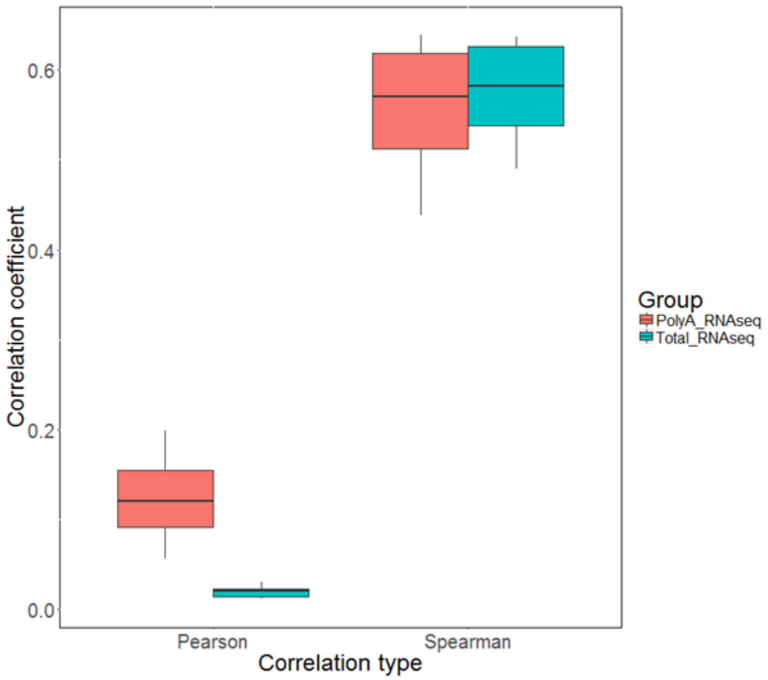

Fig. S14

Supplementary Figure 14. Correlation between promoter DHS signal and gene expression. We examined the correlation between DHS signal at promoters and gene expression levels using ENCODE cell lines for which both DHS and RNA-seq dataset were available (this included 11 cell-lines with polyA RNA-seq and 6 cell lines with total RNA-seq). In all cases, we observed high Spearman but low Pearson correlation indicating strong monotonic, non-linear relationship.

Supplementary Tables

Table S1. Number of promoter models in each regression method

Method	Data	Both	Activity level only	Binary only	None
OLS (FDR≤0.1)	ENCODE	52,658	17,807	15,437	7,007
GLM.NB(FDR≤0.1)	ENCODE	33,286	20,233	17,950	21,440
ZINB(FDR≤0.1)	ENCODE	41,336	19,919	12,672	18,982
OLS (FDR≤0.2)	ENCODE	55,975	17,083	14,036	5,815
GLM.NB(FDR≤0.2)	ENCODE	37,094	19,879	17,549	18,387
ZINB(FDR≤0.2)	ENCODE	44,240	19,742	12,384	16,543
OLS (FDR≤0.1)	Roadmap	12,315	9,526	5,242	5,546
GLM.NB(FDR≤0.1)	Roadmap	6,752	7,493	5,369	13,045
ZINB(FDR≤0.1)	Roadmap	8,728	7,646	4,550	11,705
OLS (FDR≤0.2)	Roadmap	13,124	9,530	5,053	4,922
GLM.NB(FDR≤0.2)	Roadmap	7,570	7,929	5,428	11,702
ZINB(FDR≤0.2)	Roadmap	9,520	8,064	4,566	10,479
OLS (FDR≤0.1)	FANTOM5	9,943	5,081	11,043	30,223
GLM.NB(FDR≤0.1)	FANTOM5	14,197	3,221	13,758	25,114
ZINB(FDR≤0.1)	FANTOM5	13,640	3,377	13,461	25,812
OLS (FDR≤0.2)	FANTOM5	11,072	5,127	11,503	28,588
GLM.NB(FDR≤0.2)	FANTOM5	15,396	3,210	13,530	24,154
ZINB(FDR≤0.2)	FANTOM5	14,719	3,308	13,429	24,834
OLS (FDR≤0.1)	GRO-seq	3,507	236	2,580	2,037
GLM.NB(FDR≤0.1)	GRO-seq	606	377	2,659	4,718
ZINB(FDR≤0.1)	GRO-seq	1,334	657	2,844	3,525
OLS (FDR≤0.2)	GRO-seq	3,745	249	2,509	1,857
GLM.NB(FDR≤0.2)	GRO-seq	798	453	2,830	4,279
ZINB(FDR≤0.2)	GRO-seq	1,566	681	2,907	3,206

Each promoter model contained 10 enhancers as features. The number of E-P links is $\boldsymbol{y} \cdot \mathbf{10}$ links where $\boldsymbol{y}$ is the number of promoter models in each category

Table S2. Number of statistically validated promoter models and E-P links predicted by FOCS on four genomic resources					
**Data type**	#promoter models	#*E-P links*	#Unique enhancers	% intronic E-P links *	# known genes**
**ENCODE - DHS**	70,465	167,988	92,603	74	12,256
**Roadmap - DHS**	21,841	69,619	49,327	67	10,668
**FANTOM5 - eRNA**	15,024	41,836	18,656	55	8,666
**GRO-seq - eRNA**	6,323	22,607	20,650	79	6,323

(*) E-P links whose E is located within an intron of a gene (not necessarily the target gene)
(**) Number of Entrez genes associated with promoters

Table S3. Summary of inferred E-P links				
**Method type**	**Data**	**# promoter models**	**#Links to enhancers**	**#Unique enhancers**
Pair-wise	ENCODE	92,080	2,396,287	326,184
Pair-wise-$\boldsymbol{r} = \mathbf{0.7}$	ENCODE	39,372	139,170	53,950
OLS-LASSO[1]	ENCODE	39,368	122,064	74,104
OLS-enet[1]	ENCODE	39,407	150,158	85,926
FOCS*	ENCODE	70,465	167,988	92,603
Pair-wise	Roadmap	32,000	1,023,409	106,231
Pair-wise-$\boldsymbol{r} = \mathbf{0.7}$	Roadmap	8,606	33,598	24,657
OLS-LASSO[2]	Roadmap	6,783	27,414	21,062
OLS-enet[2]	Roadmap	6,788	31,923	24,167
FOCS*	Roadmap	21,841	69,619	49,327
Pair-wise	FANTOM5	42,234	228,908	45,936
Pair-wise-$\boldsymbol{r} = \mathbf{0.7}$	FANTOM5	2,224	4,681	2,449
OLS-LASSO[3]	FANTOM5	1,680	3,970	2,219
OLS-enet[3]	FANTOM5	1,684	5,239	2,771
FOCS*	FANTOM5	15,024	41,836	18,656
Pair-wise	GRO-seq	7,825	113,817	81,040
Pair-wise-$\boldsymbol{r} = \mathbf{0.7}$	GRO-seq	4,347	26,827	24,247
OLS-LASSO[4]	GRO-seq	4,570	17,141	16,121
OLS-enet[4]	GRO-seq	4,580	21,379	19,796
FOCS**	GRO-seq	6,323	22,607	20,650
FOCS-randCV	GRO-seq	7,004	23,960	21,679

(1) The number of OLS promoter models ($\boldsymbol{R^2} \geq \mathbf{0.5}$) was 39,892 before model selection
(2) The number of OLS promoter models ($\boldsymbol{R^2} \geq \mathbf{0.5}$) was 6,807 before model selection
(3) The number of OLS promoter models ($\boldsymbol{R^2} \geq \mathbf{0.5}$) was 1,951 before model selection
(4) The number of OLS promoter models ($\boldsymbol{R^2} \geq \mathbf{0.5}$) was 4,851 before model selection
(*) Selected promoter models passed either both validation tests or the activity level test only
(**) Selected promoter models passed either binary test and/or the activity level test

Supplemental Methods

GRO-seq data preprocessing

We downloaded raw sequence data of 245 GRO-seq samples from the Gene Expression Omnibus (GEO) database (**Additional file 3: Table S5**). First, we applied read quality control on each profile using the Trimmomatic tool (default parameters) [1]. From each read we trimmed (1) bases from Illumina Tru-seq adapters, and (2) bases with low base quality scores from both ends. We excluded reads with net length <30 bases. Finally, we cropped each read to the first 30 bases from the 5' end. Second, we aligned the trimmed read to a set of known ribosomal RNA (rRNA) genes (FASTA sequences taken from NCBI: RN18S1, RN28S1, RN5, and RN5S17) using bowtie2 [2] (default parameters), and discarded reads aligned to rRNA genes. Third, we aligned the rest of the reads to hg19 reference genome using bowtie2 (default parameters). For subsequent analyses we used only reads that had a MAPQ score greater than 10. Fourth, we merged aligned reads from multiple profiles with the same sample id (via GEO GSM id) into a single sample. In total, our collected GRO-Seq database covered 40 studies encompassing 245 samples from 23 cell lines, each assayed under control and stress conditions (**Additional file 3: Table S5**).

We quantified gene transcription activity by counting the number of reads mapped within each (unspliced) gene. As gene models we used a single transcript per gene, constructed using groHMM's makeConsensusAnnotations function [3] and hg19 UCSC refGene table, producing 22,891 consensus genes. We only used reads mapped to the gene's transcript body in the range 0.5kb to 20kb downstream of the TSS. If the transcript's length was less than 20kb then we used only the region up to the transcript termination site (TTS).

To identify active enhancers in each sample, we applied dREG [4] on the aligned reads. dREG detects "*transcriptional regulation elements*" (TREs) based on symmetric forward and reverse read coverage relative to their center position. This symmetry is a known mark of short putative enhancers [5]. We merged overlapping TREs (taking the union of their locations) detected in different samples to create *merged TREs* (mTREs). We defined as enhancers mTREs that are either: (1) intergenic: mTREs whose center is located at least 5kb from the closest gene's TSS and does not overlap any gene's transcript body, or (2) intronic: mTREs that are not exonic and have overlap with an intron of a gene. We counted the number of reads in each intergenic enhancer (in both strands) and intronic enhancer (only in antisense strand) in each sample using BEDTools [6].

The gene and enhancer expression matrices were further filtered to include only genes/enhancers (rows) with at least one sample (columns) with RPKM ≥ 1, in order to preserve only expressed genes/enhancers. Next, to focus of the analysis on differential genes, we calculated for each the coefficient of variation (CoV) (the ratio between the gene's standard deviation σ to the mean μ), and selected the most variable ones as follows: (1) we partitioned the genes according to their mean RPKM expression into 20 bins. (2) In each bin we retained the

genes with CoV above the bin's median level. These two steps also reduce preference to highly or lowly expressed genes. The final gene matrix contained 8,360 genes, and the final enhancer matrix contained 255,925 enhancers.

We defined for each gene the set of k=10 candidate enhancers located within a window of ±500Kb from its TSS.

FOCS Model Implementation

The input to FOCS is two activity matrices, one for enhancers (M_e) and the other for promoters (M_p), measured across the same samples. Activity is measured by DHS signal in ENCODE and Roadmap data, and by expression level in FANTOM5 and GRO-seq data. Samples were labeled with a cell-type label out of C cell-types. The output of FOCS is predicted E-P links.

First, FOCS builds for each promoter an OLS regression model based on the k enhancers whose center positions are closest to the promoter's center position (in ENCODE, Roadmap, and FANTOM5) or TSS (in GRO-seq). Formally, let y_p be the promoter p normalized activity pattern (measured in CPM - counts per million; y_p is a row from M_p) and let X_p be the normalized activity matrix of the corresponding k enhancers (CPM; k rows from M_e). We build an OLS linear regression model $\mathrm{y_p} = \mathrm{X_p}\beta_\mathrm{p} + \varepsilon_\mathrm{p}$, where ε_p is a vector that denotes the errors of the model and β_p is the $(k+1)\ x\ 1$ vector of coefficients (including the intercept) to be estimated.

Second, FOCS performs leave-cell-type-out cross validation (LCTO CV) by training the promoter model based on samples from $C-1$ cell types and testing the predicted promoter activity of the samples from the left out cell type. This step is repeated C times. The result is a vector of predicted activity values y_p^{model} for all samples.

FOCS tests the predicted activity values using two validation tests: (1) The *binary test*. This test examines whether y_p^{model} discriminates between the samples in which p was active (observed activity $y_p \geq 1$ RPKM) and the samples in which p was inactive ($y_p < 1$ RPKM). (2) The *activity level test*. This test calculates, for the active samples, the significance of the Spearman correlation between y_p^{model} and y_p. Spearman correlation compares the ranks of the original and predicted activities. We obtain two vectors of p-values, one for each test, of length n (the number of promoter models).

Third, to correct for multiple testing, FOCS applies on each p-value vector the Benjamini - Yekutieli (BY) FDR procedure [7]. Promoter models with q-value≤ 0.1 in either both tests or in the activity level test were included in further analyses. In GRO-seq analysis, we also included models that passed only the binary test (m=2,580) since 57% of them had $R^2 \geq 0.5$ (**Fig. S6B**). For promoters that passed these CV tests final models are trained again using all samples.

FOCS next selects informative enhancers for each final promoter model. First, to control the FDR due to multiple hypotheses we used the BY correction. We call this process *enhancer BY FDR filtering* (**eBY**). The OLS results provide for each model P-values for the coefficients of its 10

closest enhancers. FOCS applies BY correction on the P-values produced by all models together and selects enhancers with q-value ≤ 0.01. To identify the most important ones out of the selected (≤ 10) enhancers for each promoter model, FOCS applies elastic-net model shrinkage (**enet**) with a regularization parameter λ, using the glmnet R function [8] with mixing parameter α=0.5, giving equal weights for Lasso and Ridge regularizations. We require that all the enhancers that survived eBY filtering will be included in the shrunken model. To achieve this we take the maximum λ satisfying this property. For models in which no enhancer survived the eBY filtering, we took the maximum λ yielding a shrunken model with at least one enhancer. This ensures that every promoter that passes the CV tests also has a model following the enet step.

Alternative regression methods

We compared the performance of OLS method with GLM.NB and ZINB regression methods. We repeated the FOCS steps but in the first step, instead of OLS we applied the GLM.NB or the ZINB methods. In GLM.NB/ZINB we used for y_p and X_p the raw count values instead of CPM. To correct the model according to differences in samples library sizes, we provided these sizes as an offset vector to GLM.NB and ZINB methods.

FANTOM5 E-P linking using OLS regression was followed by Lasso shrinkage (defined as OLS-LASSO) as described in [9]. Briefly, promoter models were created using OLS and models with $R^2 \geq 0.5$ were accepted for further analyses. Next, penalized Lasso regression was used to reduce the number of enhancers in the models. Optimal models were selected using 100-fold cross validation and the largest value of lambda such that the mean square error was within one standard error of the minimum, using the cv.glmnet() function in R glmnet package [8]. OLS followed by enet (called OLS-enet) was run with mixing parameter α = 0.5 in the cv.glmnet() function. OLS followed by LASSO (OLS-LASSO) was run with $\alpha = 1$.

GO enrichment analysis

GO enrichments were calculated using topGO R package [10] (algorithm=”classic”, statistic=”fisher”, minimum GO set size=10). We split the genes into target and background sets using their enhancer bin sets. Genes belonging to bins with 1-3/1-4/4-10/5-10 enhancers were considered as target set and compared to all genes from all bins as background set. Correction for multiple testing was performed using BH procedure [11].

External validation of predicted E-P links

We used three external data resources for validating FOCS E-P link predictions: (1) RNAPII ChIA–PET interactions, (2) YY1-HiChIP interactions, and (3) eQTL SNPs.

We downloaded 922,997 ChIA-PET interactions (assayed with RNAPII, on four cell lines: MCF7, HCT-116, K562 and HelaS3) from the chromatin–chromatin spatial interaction (CCSI) database [12] (GEO accession numbers of the ChIA-PET samples are provided in supplementary table S6). We used the liftOver tool (from Kent utils package provided by UCSC) to transform the genomic coordinates of the interactions from hg38 to hg19. HiChIP interactions mediated by YY1 TF (cell types: HCT116, Jurkat, and K562) were taken from [13] (GEO accession id: GSE99521). As done in [13], we retained 911,190 YY1-HiChIP interactions with origami probability>0.9. Origami is a method that aims to find high confident interactions. For eQTL SNPs, we used the significant SNP-gene pairs from GTEx analysis V6 and V6p builds. 2,283,827 unique eQTL SNPs covering 44 different tissues were downloaded from GTEx portal [14].

We used 1Kbp intervals (±500 bp upstream/downstream) for the promoters (relative to the center position in ENCODE/Roadmap/FNATOM5 or to the TSS position in GRO-seq) and the enhancers (±500 bp from the enhancer center). An E-P pair is considered supported by a particular capture interaction if both the promoter and enhancer intervals overlap different anchors of an interaction. An E-P pair is considered supported by eQTL SNP if the SNP is located within the enhancer's interval and is associated with the expression of the promoter's gene. For each predicted E-P pair we checked if the promoter and enhancer intervals are supported by capture interactions and eQTL data. We then measured the fraction of E-P pairs supported by these data resources.

To get an empirical P-value for the significance of the fraction, we performed 100 permutations on the data (100 permutations were sufficient as in all methods we got empirical P-value<0.01). In each permutation, for each promoter independently, if it had l E-P links, then l enhancers on the same chromosome with similar distances from the gene's TSS as the l linked enhancers were selected randomly. For this purpose we used the R 'Matching' package [15]. The fraction of overlap with the external data was computed on each permuted data.

Statistical tests, visualization and tools used

All computational analyses and visualizations were done in the R statistical language environment [16]. We used the two-sided Wilcoxon rank-sum test implemented in wilcox.test() function to compute the significance of the binary test. We used the cor.test() function to compute the significance of the Spearman correlation in the activity level test. Spearman/Pearson correlations were computed using the cor() function. To correct for multiple testing we used the p.adjust() function (method='BY'). We used 'GenomicRanges' package [17] for finding overlaps between genomic positions. We used 'rtracklayer' [18] and 'GenomicInteractions' [19] packages to import/export genomic positions. Counting reads in genomic positions was calculated using BEDTools [6]. OLS models were created using lm() function in 'stat' package[16]. GLM.NB models were created using glm.nb() function in 'MASS' package [20]. ZINB models were created using zeroinfl() function in 'pscl' package [21]. Graphs were made using graphics[16], ggplot2 [22], gplots [23], and the UCSC genome browser (https://genome.ucsc.edu/).

References

1. Bolger AM, Lohse M, Usadel B. Genome analysis Trimmomatic : a flexible trimmer for Illumina sequence data. 2014;30:2114–20.
2. Langmead B, Salzberg SL. Fast gapped-read alignment with Bowtie 2. 2012;9:357–60.
3. Chae M, Danko CG, Kraus WL. groHMM : a computational tool for identifying unannotated and cell type-specific transcription units from global run-on sequencing data. BMC Bioinformatics [Internet]. BMC Bioinformatics; 2015;9–11. Available from: http://dx.doi.org/10.1186/s12859-015-0656-3
4. Danko CG, Hyland SL, Core LJ, Martins AL, Waters CT, Lee HW, et al. Identification of active transcriptional regulatory elements from GRO-seq data. Nat. Methods. Nature Research; 2015;12:433–8.
5. Kim T-K, Hemberg M, Gray JM, Costa AM, Bear DM, Wu J, et al. Widespread transcription at neuronal activity-regulated enhancers. Nature. 2010;465:182–7.
6. Quinlan AR, Hall IM. BEDTools : a flexible suite of utilities for comparing genomic features. 2010;26:841–2.
7. Benjamini Y, Yekutieli D. The control of the false discovery rate in multiple testing under dependency. Ann. Stat. JSTOR; 2001;1165–88.
8. Friedman J, Hastie T, Tibshirani R. Regularization paths for generalized linear models via coordinate descent. J. Stat. Softw. NIH Public Access; 2010;33:1.
9. Andersson R, Gebhard C, Miguel-Escalada I, Hoof I, Bornholdt J, Boyd M, et al. An atlas of active enhancers across human cell types and tissues. Nature. 2014;507:455–61.
10. Alexa A, Rahnenfuhrer J. topGO: Enrichment Analysis for Gene Ontology. R package version 2.28.0; 2016.
11. Benjamini Y, Hochberg Y. Controlling the false discovery rate: a practical and powerful approach to multiple testing. J. R. Stat. Soc. Ser. B. JSTOR; 1995;289–300.
12. Xie X, Ma W, Songyang Z, Luo Z, Huang J, Dai Z, et al. Original article CCSI : a database providing chromatin – chromatin spatial interaction information. 2016;1–7.
13. Weintraub AS, Li CH, Zamudio A V., Sigova AA, Hannett NM, Day DS, et al. YY1 Is a Structural Regulator of Enhancer-Promoter Loops. Cell. 2017;171:1573–1579.e28.
14. Consortium Gte, others. The Genotype-Tissue Expression (GTEx) pilot analysis: Multitissue gene regulation in humans. Science (80-.). 2015;348:648–60.
15. Sekhon JS. Multivariate and propensity score matching software with automated balance optimization: the matching package for R. 2011;
16. R Core Team. R: A Language and Environment for Statistical Computing [Internet]. Vienna, Austria; 2017. Available from: https://www.r-project.org/
17. Aboyoun P, Carlson M, Lawrence M, Huber W, Gentleman R, Morgan MT, et al. Software for Computing and Annotating Genomic Ranges. 2013;9:1–10.
18. Lawrence M, Gentleman R, Carey V. rtracklayer: an R package for interfacing with genome browsers. Bioinformatics. 2009;25:1841–2.
19. Harmston, N., Ing-Simmons, E., Perry, M., et al. GenomicInteractions: R package for handling genomic interaction data [Internet]. 2015. Available from: https://github.com/ComputationalRegulatoryGenomicsICL/GenomicInteractions/
20. Venables WN, Ripley BD. Modern Applied Statistics with S. Fourth. New York; 2002.
21. Zeileis A, Kleiber C, Jackman S. Regression models for count data in R. J. Stat. Softw. 2008;27:1–25.

22. Wickham H. ggplot2: Elegant Graphics for Data Analysis [Internet]. Springer-Verlag New York; 2009. Available from: http://ggplot2.org
23. Warnes GR, Bolker B, Bonebakker L, Gentleman R, Liaw WHA, Lumley T, et al. gplots: Various R Programming Tools for Plotting Data [Internet]. 2016. Available from: https://cran.r-project.org/package=gplots
