# Supplementary material for: FOCS: a novel method for analyzing enhancer and gene activity patterns infers an extensive enhancer–promoter map
Source: Genome Biol. 2018 May 1;19:56. doi: 10.1186/s13059-018-1432-2 (PMC5930446; doi:10.1186/s13059-018-1432-2)
Supplement: Supplementary file 4 — Review history. (DOCX 473 kb) [file 13059_2018_1432_MOESM4_ESM.docx]

**First round of review**

**Reviewer 1**

**Are the methods appropriate to the aims of the study, are they well described, and are necessary controls included?**
Yes

**Are the conclusions adequately supported by the data shown?**

Yes

**Are sufficient details provided to allow replication and comparison with related analyses that may have been performed?**

Yes

**Does the method perform better than existing methods (as demonstrated by direct comparison with available methods)?**

Yes

**Is the method likely to be of broad utility? Is any software component easy to install and use?**

Yes

**Is the paper of broad interest to others in the field, or of outstanding interest to a broad audience of biologists?**

Yes: Assignment of enhancers to promoters in large metazoan genomes is an unsolved problem. This paper presents a method that attempts to solve it, and does it better than other available methods.

**Comments to authors**

The manuscript by Hait et al describes a computational approach for predicting the activity of gene promoters from the activity of nearby enhancers. The method has the potential for application to assignment of enhancers to the genes they control in situations in which the key enhancers are not the closest one to the gene and/or when the gene closest to them is not the target of the enhancer. The authors present in detail the analysis on the panel of ENCODE cell lines using DNase hypersensitivity as a measure of enhancer activity, and then repeat the approach on the data produced by alternative sources (Roadmap Epigenomics DHS) and methods (GRO-seq, CAGE from FANTOM5). They use and test three different regression methods for the purpose and find that different ones work best for different data types, use two non-parametric tests to assess the ability of enhancer states to predict the promoter state, and use the BY procedure for multiple testing correction for both. Furthermore, they use the elastic net approach to shrink the models to assess how many and which of the 10 closest enhancers are sufficient for the prediction of each promoter activity. The predictions are verified against existing 3d contact (ChIA-PET) and eQTL data, with very good results.

I would like to use the rare opportunity to commend the authors on the clarity of their writing. It is rare to read a paper of this level of technical sophistication that presents both its ideas and its methodology so clearly.

The method seems to work and produce biologically relevant predictions. I have several questions and comments, which are more about the biological assumptions an interpretation and less about the method itself.

1. When using DHS data, the authors seem to assume that DNase hypersensitivity of a promoter is monotonously growing with the increasing expression. This may be a slightly problematic assumption. DNase hypersensitivity at a promoter will depend on the type of promoter (e.g. for active promoters, CpG island-overlapping promoters are on average more DNase hypersensitive than non-CpG ones), and also some poised or bivalent promoters will be DNase hypersensitive even in situations when there is no expression from them. This way, the method might work on certain types of promoters and not on others. Could the authors check this on a hybrid set where DHS is still used as a measure of enhancer activity but a more direct measure of transcription is used for promoter activity? I would assume that the majority of ENCODE cell types with DNase data will also have RNA-seq data.

2. In the ENCODE DHS panel, the authors state that they have retained only the promoters that are active (>1RPM of DHS signal) in at least 30 out of 208 cell types. There may be bias towards a certain kind of enhancer-controlled genes. I would assume that this criterion would exclude a large number of strict tissue-specific genes, which are expressed in only one or a very small number of cell types, and favour the retention of developmentally controlled genes, i.e. those with intermediate tissue specificity, which are active in several different developmental and differentiation programmes. Could the authors provide a basic GO analysis to see for what kinds of genes does this criterion enrich, and what kinds does it tend to eliminate? Also I would expect genes expressed in fewer tissues to have fewer enhancers, so I would expect the threshold of 30/208 to have direct effect on the distributions shown in Figure 3. (A quick inspection of the prediction results indeed seems to show enrichment for developmental regulators).

3. For the set of models that passed cross validation test but have low R^2, the authors suggest that the low R^2 means that there are additional missing regulatory elements that play important role in the regulation of the target promoter. This suggests that these promoters will on average be under much longer-range regulation than the majority of others. Since it is known that this set of target genes is enriched for genes that encode developmental transcription factors and other developmental regulators, a quick GO analysis of these genes should provide additional evidence that low R^2 is indeed due to additional enhancers further away from the closest 10.

4. A minor point: the authors use the term "architecture" to mean something different than it normally means to promoter researchers, for whom promoter architecture refers to the configuration of general transcription factors and nucleosomes and where different architectures point to qualitatively different classes of promoters. I would suggest to find an alternative term for their "architecture of promoter regulation by enhancers" ("arrangement" or "configuration" may work).

**Reviewer 2**

**Are the methods appropriate to the aims of the study, are they well described, and are necessary controls included?**

Yes

**Are the conclusions adequately supported by the data shown?**

Yes. Functional tests of some of the promoter-enhancer links would have been great but probably outside the scope of this paper.

**Are sufficient details provided to allow replication and comparison with related analyses that may have been performed?**

No. The users should make it easier to attain the data used in the paper (provide code for data download). I tried to get some of the data but it was unavailable. See the full review.

**Is the method likely to be of broad utility? Is any software component easy to install and use?**

It is of broad utility. I wasn't able to test it due to lack of data availability. It could definitely be made easier to use. The tutorial was just an R script. I think an accompanying text file or webpage describing the scripts and showing example usage would be helpful.

**Is the paper of broad interest to others in the field, or of outstanding interest to a broad audience of biologists?**

Yes, connecting enhancers to promoters of great interest both to those studying gene regulation and to biologist trying to understand the impact of non-coding variation.

**Comments to authors**

The submitted manuscript "FOCS: A novel method for analyzing enhancer and gene activity patterns infers an extensive enhancer-promoter map" describes a new method for inferring enhancer-promoter relationships. Their method corrects for many sources of bias and error. They compare inferences made by FOCS to existing methods as well as to chromatin contact and eQTL data sets showing both increased sensitivity and accuracy. The paper is well written and easy to understand. To the best of my knowledge the FOCS method is based on sound statistical principles. While I cannot explicitly comment on novelty, I believe this is a advance to the field. With moderate revision I believe this will be appropriate for publication in Genome Biology.

Major Points

1) While the approach is sound, I cannot comment on novelty. The FOCS methods uses cross-validation to reduce over fitting, non parametric tests to allow for non-linear relationships, and methods that are less sensitive to outliers and zeros. These are all, at least in principle, important aspects for this type of analysis and a major improvement over older methods that simply rely on Pearsons correlations. The papers the authors cite as relying on Pearsons are somewhat old though (2012 and 2014). I am not intimately familiar with this field but I would have thought there were more recent papers using more advanced statistics. By performing literature searches I found extensive work in this area, although some of it uses other data types as well (ChIP-seq, segmentation, etc) and therefore may not qualify. Hopefully other reviewers can comment on novelty (if this is important to the editors).

2) The enhancer distance is an interesting part of the paper that the authors should explore more deeply. (A) While I understand the need to limit the number of enhancers per promoter, I am also somewhat concerned about what biases this might introduce. For some promoters the 10th ranked enhancer may be 50 Kb away, whereas for others it may be 500Kb. It might be interesting to how many promoters fall into these categories. (B) The plot in Fig 3D is interesting. But I would also like to see that plot made using distance bins instead of proximity ranking. I understand why you have to filter for the closest 10, but there is not reason the data needs to be plotted that way. (C) In addition to the 10 enhancers, I think a great control would be to add another enhancer from a different chromosome to each model. Would the inclusion frequency be 0? Or would it be closer to the plateau in fig 3D. This would provide a valuable insight into false positive rates.

3) The plots in the figures were well chosen to convey the critical information but could use improvement for clarity (especially figure 5). Figure 1, in particular was very helpful. Many plots could use better legends/labeling. For example, the colors in Fig 2E are described in the caption but could be easily added as a legend in the plot. There is even some white space to use in the top left. Figure 5, was very confusing. The text on the left of the plot is not aligned, and might be different sizes. It was hard to see what was being labeled. The 'bar bells' in the bottom left of each plot were confusing. Those should be made to look more link the links in the plot itself. There is just a lot of text floating around different parts of the plot. It took awhile to figure out what was going on. Reorganization of this plot would not take long but would significantly improve interpretation. If the authors use in situ HiC data (as suggested in comment 5 below) they could put the contact matrix above the signal tracks. This would allow the reader to see how the actual contact frequencies correlate with the FOCS E-P inferences and would be much more informative than the lines representing ChIA-PET interactions.

4) We suggest that the authors provide the commands used to download the data used in these analyses. It is important that the authors distribute the code used to run the analyses which they did. However, the authors do not provide the actual data sets, nor describe succinctly how to attain them, making it difficult for me to review the paper/method. For example, I tried to get the ChIA-PET data that they used. However, the link to the CCSI database was not working. I suggest that the authors provide the commands to download the data or provide the data themselves. While I do not think the authors need to drastically change their scripts, it would also be helpful to add more comments describing certain steps (particularly those steps that require the user to modify, e.g. path variables).

5) I strongly suggest that the authors use in situ Hi-C data to validate their results instead of ChIA-PET data. It is great that the authors validated their approach using existing data sets but I do question the quality of the ChIA-PET data set. Since the link to the CCSI database was not working I am not sure exactly which ChIA-PET data sets were used (which is also a problem). But the fact that they contained almost a million interactions suggests that they were not processed correctly. It is currently pretty well accepted that there are only ~10,000 specific interactions (i.e. loops) per cell type. Many ChIA-PET data sets (especially older ones) were not processed carefully and exhibit extremely high false positive rates. The majority of interactions reported in those data sets are (somewhat) random subsets of intra TAD interactions, and not specific contacts between two loci. Even very well processed ChIA-PET data sets struggle to distinguish between loops and non-specific intra TAD contacts. Rao et al (Cell 2014) provides high quality loop calls for 8 human cell lines. Each line has <= 10,000 loops which is currently thought to be the correct order of magnitude. This data is much more reliable.

Minor points
There were a lot of typos. The reference to Fig 1A-B on line 288 ( 2nd line on page 13) seems wrong.

**Authors’ response to reviewers**

***Reviewer #1****:*

*The manuscript by Hait et al describes a computational approach for predicting the activity of gene promoters from the activity of nearby enhancers. The method has the potential for application to assignment of enhancers to the genes they control in situations in which the key enhancers are not the closest one to the gene and/or when the gene closest to them is not the target of the enhancer. The authors present in detail the analysis on the panel of ENCODE cell lines using DNase hypersensitivity as a measure of enhancer activity, and then repeat the approach on the data produced by alternative sources (Roadmap Epigenomics DHS) and methods (GRO-seq, CAGE from FANTOM5). They use and test three different regression methods for the purpose and find that different ones work best for different data types, use two non-parametric tests to assess the ability of enhancer states to predict the promoter state, and use the BY procedure for multiple testing correction for both. Furthermore, they use the elastic net approach to shrink the models to assess how many and which of the 10 closest enhancers are sufficient for the prediction of each promoter activity. The predictions are verified against existing 3d contact (ChIA-PET) and eQTL data, with very good results.*

*I would like to use the rare opportunity to commend the authors on the clarity of their writing. It is rare to read a paper of this level of technical sophistication that presents both its ideas and its methodology so clearly.*

We thank the reviewer for the compliment. We made great efforts to describe our method and analyses in a way that will be accessible to a broad readership. We are heartened that the reviewer finds that we met this challenge well.

*The method seems to work and produce biologically relevant predictions. I have several questions and comments, which are more about the biological assumptions and interpretation and less about the method itself.*

1. *When using DHS data, the authors seem to assume that DNase hypersensitivity of a promoter is monotonously growing with the increasing expression. This may be a slightly problematic assumption. DNase hypersensitivity at a promoter will depend on the type of promoter (e.g. for active promoters, CpG island-overlapping promoters are on average more DNase hypersensitive than non-CpG ones), and also some poised or bivalent promoters will be DNase hypersensitive even in situations when there is no expression from them. This way, the method might work on certain types of promoters and not on others. Could the authors check this on a hybrid set where DHS is still used as a measure of enhancer activity but a more direct measure of transcription is used for promoter activity? I would assume that the majority of ENCODE cell types with DNase data will also have RNA-seq data.*

We thank the reviewer for this important point. We examined the RNA-seq data provided by ENCODE. Somewhat surprisingly, such data is available for only a fraction of the cell lines: only 11 cell lines have polyA RNA-seq and 6 cell lines have total RNA-seq. Given this low number, we could not robustly test FOCS on a hybrid set as suggested. Our analysis of DHS data examines how well promoter DHS signal is predicted by its enhancers DHS signal. The biological interpretation of the results indeed implicitly assumes a monotonic relationship between promoter DHS signal and transcriptional activity. In response to the reviewer's question, we examined DHS-expression correlation in each ENCODE cell line for which both datasets are available. In all cases, we observed high Spearman but low Pearson correlation (see figure below), indicating strong monotonic but non-linear relationship. We discuss this issue in the revised Discussion and added the figure as Supplementary Figure 14.

Of note, the 'activity-level validation' test implemented in FOCS applies Spearman (rather than Pearson) correlation test, which fits the case of monotonic non-linear E-P relationships.

*2. In the ENCODE DHS panel, the authors state that they have retained only the promoters that are active (>1RPM of DHS signal) in at least 30 out of 208 cell types. There may be bias towards a certain kind of enhancer-controlled genes. (1) I would assume that this criterion would exclude a large number of strict tissue-specific genes, which are expressed in only one or a very small number of cell types, and favour the retention of developmentally controlled genes, i.e. those with intermediate tissue specificity, which are active in several different developmental and differentiation programmes? (2) Could the authors provide a basic GO analysis to see for what kinds of genes does this criterion enrich, and what kinds does it tend to eliminate? (3) Also I would expect genes expressed in fewer tissues to have fewer enhancers, so I would expect the threshold of 30/208 to have direct effect on the distributions shown in Figure 3. (A quick inspection of the prediction results indeed seems to show enrichment for developmental regulators).*

(1) By design our method is based on the conservative leave cell-type out cross validation, and therefore is indeed intended for delineating enhancers that regulate genes with intermediate tissue specificity and does not account for genes whose regulation is strictly cell-type specific. We have added an explanation to the Discussion on this aspect of our approach. (2) As the reviewer suggested we performed GO analysis on the genes that were filtered out/kept by our filtering scheme (Number of kept and filtered out Entrez genes: 13,141 and 828, respectively). As expected, the filtered out genes tend to be enriched in highly tissue-specific processes, while, since all house-keeping genes are kept, the most highly enriched GO categories for the kept genes are related to basic functions common to all cell types. Below are representative GO terms for these two gene sets:

For the filtered out set:

**GO.ID	Term	# Annotated	# tarSet	Expected	classicFisher	qval**

GO:0050877	neurological system process	613	88	35.7	1.30E-15	3.16E-12

GO:0007600	sensory perception	342	59	19.92	3.00E-14	3.55E-11

GO:0099537	trans-synaptic signaling	465	59	27.08	1.10E-08	2.64E-06

GO:0002250	adaptive immune response	259	39	15.09	4.00E-08	9.51E-06

GO:0007586	digestion	83	18	4.83	1.00E-06	0.000165

For the kept set:

**GO.ID	Term	# Annotated	# tarSet	Expected classicFisher	qval**

GO:0044237	cellular metabolic process	8132	7771	7658.35	5.20E-19	5.08E-16

GO:1903047	mitotic cell cycle process	809	800	761.88	1.70E-12	4.14E-10

GO:0006396	RNA processing	765	756	720.44	1.70E-11	2.74E-09

GO:0010468	regulation of gene expression 3565	3427	3357.36	6.20E-10	7.58E-08

GO:0051641	cellular localization	2148	2079	2022.89	8.80E-10	1.02E-07

(3) We thank the reviewer for the interesting hypothesis on a possible relationship between the breadth of gene expression across tissues and number of regulating enhancers. In fact, the results we showed for house-keeping genes (Supplementary Fig 11 in the revised ms), which have maximal expression breadth and a simple regulatory logic, reflected by low number of enhancers, suggested that the link might be more intricate. We further explored this point, applying the commonly used Shannon entropy as a measure for expression breadth (higher entropy indicates higher tissue span). As shown in the figure below, we find an inverse relationship: more tissuerestricted promoter activity profile (lower entropy) is associated with higher number of linked enhancers. We include this result in the revised manuscript (Fig. 6 and Supplementary Figure 12).

Interestingly, while we found no enriched GO categories for genes assigned to bins with low number of linked enhancers, several categories related to cell communication and differentiation were enriched among the genes with higher number of linked enhancers. Below are selected GO terms that were overrepresented when analyzing together the genes with 4-10 linked enhancers. We added these results to the revised manuscript (Supplementary Table 4).

**GO.ID	Term	# Annotated	# tarSet	Expected classicFisher	qval**

GO:0007155	cell adhesion	902	186	115.41	3.70E-12	1.03E-08

GO:0007165	signal transduction	3626	557	463.94	1.30E-08	8.26E-06

GO:0006954	inflammatory response	388	83	49.64	1.10E-06	0.000296

GO:0030154	cell differentiation	2505	371	320.51	0.00039	0.017444

*3. For the set of models that passed cross validation test but have low R^2, the authors suggest that the low R^2 means that there are additional missing regulatory elements that play important role in the regulation of the target promoter. This suggests that these promoters will on average be under much longer-range regulation than the majority of others. Since it is known that this set of target genes is enriched for genes that encode developmental transcription factors and other developmental regulators, a quick GO analysis of these genes should provide additional evidence that low R^2 is indeed due to additional enhancers further away from the closest 10.*

Here we chose to perform a gene-set enrichment analysis (GSEA) on the genes ranked by R^2 rather than GO analysis, as GSEA is generally more sensitive and does not require specifying a threshold. No geneset (either function or biological process category or signaling pathway) was enriched among the genes with low R^2, so we could not associate these genes with any specific function/process. We did observe some enrichment for the genes with high R^2, including immune-related genes, Notch1 targets and collagens, as shown in the figure below. We decided not to include these results as we are not sure about their biological meaning.

*4. A minor point: the authors use the term "architecture" to mean something different than it normally means to promoter researchers, for whom promoter architecture refers to the configuration of general transcription factors and nucleosomes and where different architectures point to qualitatively different classes of promoters. I would suggest to find an alternative term for their "architecture of promoter regulation by enhancers" ("arrangement" or "configuration" may work).*

Thanks for this suggestion. We replaced "architecture" by "configuration".

***Reviewer #2****:*

*The submitted manuscript "FOCS: A novel method for analyzing enhancer and gene activity patterns infers an extensive enhancer-promoter map" describes a new method for inferring enhancer-promoter relationships. Their method corrects for many sources of bias and error. They compare inferences made by FOCS to existing methods as well as to chromatin contact and eQTL data sets showing both increased sensitivity and accuracy. The paper is well written and easy to understand. To the best of my knowledge the FOCS method is based on sound statistical principles. While I cannot explicitly comment on novelty, I believe this is a advance to the field. With moderate revision I believe this will be appropriate for publication in Genome Biology.*

*Major Points*

*1) While the approach is sound, I cannot comment on novelty. The FOCS methods uses cross-validation to reduce over fitting, non parametric tests to allow for non-linear relationships, and methods that are less sensitive to outliers and zeros. These are all, at least in principle, important aspects for this type of analysis and a major improvement over older methods that simply rely on Pearsons correlations. The papers the authors cite as relying on Pearsons are somewhat old though (2012 and 2014). I am not intimately familiar with this field but I would have thought there were more recent papers using more advanced statistics. By performing literature searches I found extensive work in this area, although some of it uses other data types as well (ChIP-seq, segmentation, etc) and therefore may not qualify. Hopefully other reviewers can comment on novelty (if this is important to the editors).*

While our manuscript was under review, a related work that also applies an advanced statistical method (implemented in a tool called *JEME*) for E-P mapping was just published (*Nat Genet. 2017 Oct;49(10):1428-1436*). We mention and cite this study, as well as TargetFinder that was previously cited, in the revised Discussion.

*2) The enhancer distance is an interesting part of the paper that the authors should explore more deeply.*

*(A) While I understand the need to limit the number of enhancers per promoter, I am also somewhat concerned about what biases this might introduce. For some promoters the 10th ranked enhancer may be 50 Kb away, whereas for others it may be 500Kb. It might be interesting to show how many promoters fall into these categories. (B) The plot in Fig 3D is interesting. But I would also like to see that plot made using distance bins instead of proximity ranking. I understand why you have to filter for the closest 10, but there is no reason the data needs to be plotted that way.*

We thank the reviewer for this point. Below please find (i) a plot for the E-P distance distribution of the all enhancers that were considered by FOCS. (ii) The distance distribution of the 10th ranked enhancer and (iii) a plot for enhancer inclusion rate as a function of its distance. We added these plots to the revised manuscript (Supplementary Figure 2 and 8).

*(C) In addition to the 10 enhancers, I think a great control would be to add another enhancer from a*

*different chromosome to each model. Would the inclusion frequency be 0? Or would it be closer to the*

*plateau in fig 3D. This would provide a valuable insight into false positive rates.*

We thank the reviewer for this excellent idea. We implemented this suggestion and randomly selected 10,000 promoter models from the 70,465 models that passed the CV step. For each model, we created a modified model that included the original 10 enhancers + one enhancer that was randomly selected from a different chromosome. When applying enet to these 10,000 modified modes, only 7 retained the 11th enhancer. This inclusion frequency (0.0007) is significantly lower than the plateau reached in Fig. 3D and the rate shown by all distance bins (the plot inserted above which is included in the revised ms as Supplementary Figure 2C). This result indicates low false positive rate among the enhancers included in the final model after enet selection for all E-P distance bins. We included this control experiment in the revised manuscript (Results, page 11).

*3) The plots in the figures were well chosen to convey the critical information but could use improvement for clarity (especially figure 5). Figure 1, in particular was very helpful. Many plots could use better legends/labeling. For example, the colors in Fig 2E are described in the caption but could be easily added as a legend in the plot. There is even some white space to use in the top left.*

We improved Fig 2E and added the caption to the plot itself.

*Figure 5, was very confusing. The text on the left of the plot is not aligned, and might be different sizes. It was hard to see what was being labeled. The 'bar bells' in the bottom left of each plot were confusing. Those should be made to look more like the links in the plot itself. There is just a lot of text floating around different parts of the plot. It took awhile to figure out what was going on. Reorganization of this plot would not take long but would significantly improve interpretation. If the authors use in situ HiC data (as suggested in comment 5 below) they could put the contact matrix above the signal tracks. This would allow the reader to see how the actual contact frequencies correlate with the FOCS E-P inferences and would be much more informative than the lines representing ChIA-PET interactions.*

We reorganized Figure 5 and hope the reviewer finds the new version less cluttered and clearer.

*4) We suggest that the authors provide the commands used to download the data used in these analyses. It is important that the authors distribute the code used to run the analyses which they did. However, the authors do not provide the actual data sets, nor describe succinctly how to attain them, making it difficult for me to review the paper/method. For example, I tried to get the ChIA-PET data that they used. However, the link to the CCSI database was not working. I suggest that the authors provide the commands to download the data or provide the data themselves. While I do not think the authors need to drastically change their scripts, it would also be helpful to add more comments describing certain steps (particularly those steps that require the user to modify, e.g. path variables).*

We understand that the reviewer later could locate data (see opening of the Editor's letter). We now

specify the data location more clearly and provide GEO accession numbers of the original datasets

(Methods and Supplementary Tables 5-6). We also provide the code to all our scripts both through our

website and through github and zenodo repositories. In addition, we wrote a very detailed tutorial

http://acgt.cs.tau.ac.il/focs/tutorial.html and provide links for downloading the datasets we used

<http://acgt.cs.tau.ac.il/focs/download.html>

*5) I strongly suggest that the authors use in situ Hi-C data to validate their results instead of ChIA-PET data. It is great that the authors validated their approach using existing data sets but I do question the quality of the ChIA-PET data set. Since the link to the CCSI database was not working I am not sure exactly which ChIA-PET data sets were used (which is also a problem). But the fact that they contained almost a million interactions suggests that they were not processed correctly. It is currently pretty well accepted that there are only ~10,000 specific interactions (i.e. loops) per cell type. Many ChIA-PET data sets (especially older ones) were not processed carefully and exhibit extremely high false positive rates. The majority of interactions reported in those data sets are (somewhat) random subsets of intra TAD interactions, and not specific contacts between two loci. Even very well processed ChIA-PET data sets struggle to distinguish between loops and non-specific intra TAD contacts. Rao et al (Cell 2014) provides high quality loop calls for 8 human cell lines. Each line has <= 10,000 loops which is currently thought to be the correct order of magnitude. This data is much more reliable.*

We downloaded E-P links inferred from Hi-C data of Rao et al. (GSE63525), collectively including

43,219 loops in 8 cell types (GM12878, HeLa, HMEC, HUVEC, IMR90, K562, KBM7, NHEK). The loops analyzed in these dataset are of a different scale than ours: The minimum distance between midpoints of loop anchors is 35kb, the median is ~250kb and the average is 669kb. In contrast, the majority of E-P links inferred by FOCS is below 10kb. As a result, the overlap is very low (less than 1%). Similarly, the overlap between E-P links inferred by Pearson pairwise correlation and the Rao et al. loops is ~2%. To remedy this, we tried to use E-P links inferred from these data by PSYCHIC, a more advanced computational method (Nature Comm. 2017, PMID: 29269730 ; 161,029 E-P links at q-value≤1e-2). Only ~7% of the links predicted by either FOCS or Pearson pairwise correlation were supported by PSYCHIC links. This implies that the current resolution of Hi-C may be insufficient for using it to validate predicted E-P links.

We therefore sought another data source for validating FOCS predictions. Very recently, Weintraub et al. (Cell. 2017 Dec 14;171(7):1573-1588) performed YY1-HiChIP experiments in 3 cell lines (HCT116, Jurkat, K562) and demonstrated that YY1 acts as a general structural regulator of E-P loops. We therefore used these data (a total of 911,190 YY1-HiChIP interactions with origami score >0.9) for benchmarking. While 36.6% of FOCS E-P links are supported by these YY1-HiChIP interactions, only 7.4% of the Pearson pairwise E-P links are supported (and 11.3% of the Pearson-pairwise E-P links with R>0.7). FOCS predictions also got markedly higher support rate on E-P links inferred from the Roadmap Epigenomics and GRO-seq data. Support rate for FOCS prediction was lower for the FANTOM5 data (as we observed and reported before using RNAPII ChIA-PET data). We included these results in the revised manuscript (Fig. 4 and Supplementary Figure 9).

As a side note, regarding the estimated number of E-P loops per cell, we believe that 10,000 is an underestimate. Considering ~10,000 expressed protein-coding genes and a similar number of noncoding genes gives a total of ~20,000 active promoters. On average, each promoter is associated with ~3-4 enhancers. So the number of E-P links in a cell is probably closer to 50,000-100,000 interactions. On top of these, ChIA-PET also detects multiple P-P and E-E loops. Time will tell which estimates are closer to the actual figures.

*Minor points*

*There were a lot of typos. The reference to Fig 1A-B on line 288 ( 2nd line on page 13) seems wrong.*

We have carefully proofread the entire manuscript. The reference on page 13 was indeed wrong and is

now corrected (to Fig 2A-B).

**Second round of review**

**Reviewer 1**

The authors have responded adequately to my original remarks.

**Reviewer 2**

The authors did a good job of responding to all of our comments. They responded to them in text, reanalyzed their data, added extra figures, and wrote very clear responses. I recommend this paper for publication but also have two comments below:

1) Figure 5 could still be labeled a little clearer. If you do nothing else, please replace the barbells in the bottom left corner with links that look more like the links in the plots themselves (same stroke width etc). It should only take 60 seconds to change and will make it much easier for the readers to understand.

2) I commend the authors for taking an honest look at Hi-C data sets and even adding an analysis with Hi-ChIP data. It is true that well processed Hi-C struggles to identify loops smaller than ~25Kb. But I have seen no evidence that ChIA-PET or Hi-ChIP does any better. While I have serious questions regarding the validity of these loop calls, I cannot suggest a better set of loop calls for such short distances. At this point, I would suggest that authors add a statement along the lines of “While 3C-based methods are generally not well equipped to identify DNA loops below 25Kb, we intersected our results with the best available loop calls for these data ranges”.

In summary, the FOCS method appears to be a solid advancement in our ability to connect enhancers to their promoters and predict expression. The method is solid, the writing is clear, and I believe that this manuscript is suitable for publication in Genome Biology.
